# Cell-selective labelling of proteomes in *Drosophila melanogaster*

**DOI:** 10.1038/ncomms8521

**Published:** 2015-07-03

**Authors:** Ines Erdmann, Kathrin Marter, Oliver Kobler, Sven Niehues, Julia Abele, Anke Müller, Julia Bussmann, Erik Storkebaum, Tamar Ziv, Ulrich Thomas, Daniela C. Dieterich

**Affiliations:** 1 Research Group Neuronal Plasticity and Communication, Institute for Pharmacology and Toxicology, Otto-von-Guericke-University Magdeburg, Magdeburg 39120, Germany; 2 Research Group Neuralomics, Leibniz Institute for Neurobiology, Magdeburg 39118, Germany; 3 Department of Neurochemistry and Molecular Biology, Leibniz Institute for Neurobiology, Magdeburg 39118, Germany; 4 Molecular Neurogenetics Laboratory, Max Planck Institute for Molecular Biomedicine, Münster 48149, Germany; 5 Faculty of Medicine, University of Münster, Münster 48149, Germany; 6 Smoler Proteomics Center, Faculty of Biology, Technion, Haifa 32000, Israel; 7 Center for Behavioral Brain Sciences, Magdeburg 39118, Germany

## Abstract

The specification and adaptability of cells rely on changes in protein composition. Nonetheless, uncovering proteome dynamics with cell-type-specific resolution remains challenging. Here we introduce a strategy for cell-specific analysis of newly synthesized proteomes by combining targeted expression of a mutated methionyl-tRNA synthetase (MetRS) with bioorthogonal or fluorescent non-canonical amino-acid-tagging techniques (BONCAT or FUNCAT). Substituting leucine by glycine within the MetRS-binding pocket (MetRS^LtoG^) enables incorporation of the non-canonical amino acid azidonorleucine (ANL) instead of methionine during translation. Newly synthesized proteins can thus be labelled by coupling the azide group of ANL to alkyne-bearing tags through ‘click chemistry'. To test these methods for applicability *in vivo*, we expressed MetRS^LtoG^ cell specifically in *Drosophila*. FUNCAT and BONCAT reveal ANL incorporation into proteins selectively in cells expressing the mutated enzyme. Cell-type-specific FUNCAT and BONCAT, thus, constitute eligible techniques to study protein synthesis-dependent processes in complex and behaving organisms.

## Abstract (web summary)

Mutated tRNA synthetases can incorporate non-canonical amino acids into proteins. Erdmann *et al.* exploit this property to metabolically label newly synthesized proteins in selected cell types in *Drosophila*, and demonstrate their detection using proteomics (BONCAT) and fluorescence imaging (FUNCAT).

## Graphical abstract

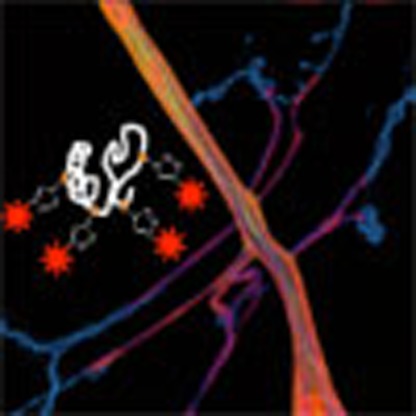

Cell type diversification and the execution of cell-type-specific functions is tightly coupled with the establishment of cell-specific proteomes and their proper modulation according to extrinsic and intrinsic signals. In fact, regulatory processes that act on protein synthesis, degradation and post-translational modification are permissive and sometimes instructive to higher-order organismal functions such as the adaptation of energy metabolism, immune defence or the acquisition and storage of memory. Thus, characterizing cell-specific proteome dynamics is key to understand physiological and pathological conditions at the level of single cells, organs and, ultimately, whole organisms. Facing ∼10,000 different proteins in a mammalian cell^1^, in-depth identification of such a cell's proteome, let alone the comparison with other cellular proteomes, is highly challenging. Proteome characterizations become even more demanding if temporal and spatial aspects, that is, proteome dynamics, are to be considered. Immense technological advances have allowed for increasingly efficient assessments of proteomes of eukaryotic cell lines, distinct tissues and readily isolatable cell types such as blood cells or sperm^2^,^3^,^4^,^5^. Moreover, refined methods for protein fractionation and enrichment according to various criteria have led to successful characterizations of cellular subproteomes. For instance, post-translational modifications such as phosphorylation or ubiquitination provide a suitable handle for enrichment of the ‘phosphoproteome'^6^ or for proteins destined for degradation via ubiquitination^7^ or sumoylation^8^. In contrast, reducing sample complexity by selectively enriching for newly synthesized proteins is troublesome, since all proteins—old and new—share the same pool of 20 amino acids. Thus, until now, monitoring proteomes in multicellular organisms with cell-type-specific and temporal resolution has been impossible for cells that are tightly embedded in their respective tissues. As another complication, cellular and subcellular proteome turnover rates are usually low as recently demonstrated for the pool of synaptic proteins in mammalian primary cultures with a turnover rate of 0.7% of the synaptic protein content per hour^9^.

Bioorthogonal labelling strategies have been developed to globally label newly synthesized proteins or distinct post-translational modifications using the endogenous biosynthesis machinery (reviewed in ref. 10). Global metabolic labelling of newly synthesized proteins can be achieved using isotopic amino acids (as used in SILAC in combination with mass spectrometry^11^), functionalized non-canonical amino acids^12^ or functionalized derivatives of the protein synthesis inhibitor puromycin^13^. The non-canonical amino acids azidohomoalanine (AHA) or homopropargylglycine (HPG) harbor an azide or an alkyne group, respectively, and these groups confer unique chemical functionality to their target molecules, which can subsequently be tagged with exogenously delivered probes for detection or isolation in a highly selective manner using copper-catalyzed [3+2] azide-alkyne-cycloaddition (CuAAC, ‘click chemistry'). AHA or HPG is used instead of methionine by the endogenous methionyl-transfer RNA (tRNA) synthetase (MetRS), that is, loaded onto respective tRNAs and incorporated into nascent polypeptide chains. The new chemical functionality of these proteins can be exploited for their identification (BONCAT, bioorthogonal non-canonical amino-acid tagging^14^,^15^) and visualization (FUNCAT, fluorescent non-canonical amino-acid tagging^16^,^17^,^18^). Both methods enable not only the analysis of activity-dependent global changes of cellular proteomes but also the identification of local synthesis hot spots in cellular subcompartments such as dendrites^17^. So far, AHA and HPG have been used to track new protein synthesis in a variety of model systems including bacteria^19^, mammalian cell culture models^16^,^17^,^20^,^21^ and larval zebrafish^22^.

Recently, Tirrell and co-workers reported mutated variants of bacterial MetRS (EcMetRS), which charge the azide-harbouring amino acid azidonorleucine (ANL) onto methionine initiator tRNA, allowing its N-terminal incorporation into nascent proteins^23^,^24^. Importantly, in complex bacterial or mammalian cell mixtures, the residue-specific incorporation of ANL was restricted to cells expressing a mutant MetRS^25^,^26^.

The fruit fly *Drosophila* has recently been used in a number of studies that addressed subproteomes as diverse as the networks of proteins involved in the Hippo signalling pathway, innate immune reactions or the assembly of presynaptic active zones^27^,^28^,^29^. Moreover, high-coverage proteome analyses have been performed for *Drosophila* S2-cells and for different developmental stages of the organism, which resulted in valuable reference data sets, for example, for establishing comprehensive protein–protein interaction maps^30^,^31^. Here we present a method that adds cell selectivity as an important expansion for labelling proteomes in living *Drosophila* larvae and adult flies. A crucial step was to identify a leucine to glycine substitution within the binding pocket of murine or *Drosophila* MetRS (L274G or L262G, respectively; collectively referred to as MetRS^LtoG^) as a way to enable the enzymes to charge *Drosophila* methionyl-tRNA with ANL. On targeted expression of MetRS^LtoG^ in flies, efficient cell-specific, time-dependent and concentration-dependent incorporation of food-supplied ANL into proteins is uncovered by FUNCAT. Moreover, BONCAT reveals a bulk number of labelled proteins in each of the tested cell types, implying that many proteins can be assessed simultaneously for cell-type-specific, temporally controlled and context-dependent synthesis of proteins.

## Results

### *Drosophila* proteomes are amenable to tagging by AHA

As previously shown, the non-canonical amino acid AHA (Supplementary Fig. 1a) can be used to detect newly synthesized proteins^10^. Therefore, to assess the principal amenability of *Drosophila* for metabolic protein labelling by FUNCAT and BONCAT, we grew larvae and adult flies on AHA-containing food and subjected them to either type of analysis as schematically depicted in Supplementary Fig. 1b. Using the red-fluorescent dye tetramethylrhodamine (TAMRA) in CuAAC reactions on larval body walls revealed efficient incorporation of AHA into muscles (Supplementary Fig. 2a) and any other tissue attached to this preparation such as motor neurons. Only low-background fluorescence levels were monitored when the reaction was applied to animals grown on AHA-free food (Supplementary Fig. 2b). Consistently, CuAAC-mediated coupling of a biotin-alkyne tag was efficient and specific for protein samples isolated from AHA-fed flies as revealed by western blot analysis (see accompanying paper by Niehues *et al.*, Supplementary Fig. 10b).

### Cell-specific *in situ* labelling of proteins by NCAT

**Figure 1:**
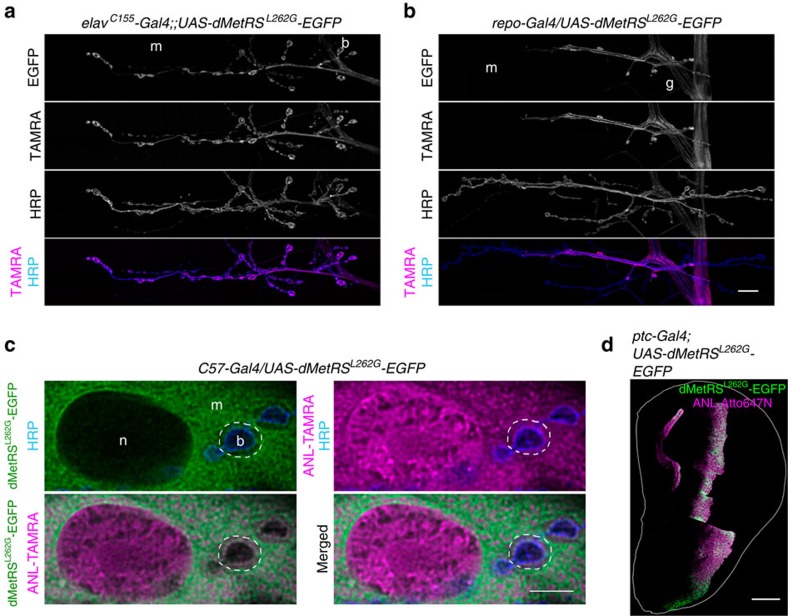
Cell-type-specific *in situ* labelling of proteins via FUNCAT. (**a**–**c**) ANL incorporation into larval proteins is monitored via FUNCAT on targeted expression of dMetRS^L262G^-EGFP in neurons (**a**, *elav*^*C155*^*-Gal4;;UAS-dMetRS*^*L262G*^*-EGFP*), glia cells (**b***, repo-Gal4/UAS-dMetRS*^*L262G*^*-EGFP*) and muscle cells (**c**, *C57-Gal4/UAS-dMetRS*^*L262G*^*-EGFP*) at larval neuromuscular junctions (muscles 6/7, segment A2). Co-staining with the neuron-specific marker anti-HRP (**a**–**c**) reveals that, wherever nerve terminal boutons (b), glial protrusions (g) and muscles (m) are in close contact, ANL-TAMRA signals are restricted to the dMetRS^L262G^-EGFP-expressing cell type (**a**–**c**). dMetRS^L262G^-EGFP is predominantly found in the cytosol, whereas TAMRA-harbouring proteins are detectable throughout cells including nuclei (n) and the bouton surrounding SSR area (**c**, dashed line). (**d**) Expression of dMetRS^L262G^-EGFP in a wing disc epithelium along the anterior–posterior border (*ptc-Gal4;UAS-dMetRS*^*L262G*^*-EGFP*) is accompanied by a respective confinement of ANL-Atto647N signals. The outline represents the shape of the entire disc. Scale bars, 10 μm (**a**,**b**); 5 μm (**c**); and 50 μm (**d**). SSR, subsynaptic reticulum.

Next, we replaced AHA in the food by the non-canonical amino acid ANL (Supplementary Fig. 1a). Neither FUNCAT nor BONCAT assays revealed incorporation of ANL into proteomes of wild-type flies (Supplementary Fig. 2c), implying that endogenous aminoacyl-tRNA synthetases are unable to use ANL as a substrate. Previous work, however, revealed that various mutations altering the methionine-binding pocket of the MetRS from *Escherichia coli* (EcMetRS) enabled the enzyme to couple ANL to Met-tRNA and that the product is efficiently used in protein synthesis^23^,^24^,^25^,^26^,^32^. Among those mutant MetRS forms are the single amino acid mutant EcMetRS^L13G^ and the triple amino acid mutant EcMetRS^NLL^. The binding pocket of MetRS is evolutionary well-conserved (Supplementary Fig. 3) and this led us to construct enhanced green fluorescent protein (EGFP)-tagged forms of wild type and mutated murine MetRS (mMetRS). As the mMetRS with the single amino-acid mutation L274G incorporated ANL most efficiently in cell culture assays (A.M. and D.C.D., unpublished observations), it was used to generate flies carrying the Gal4-inducible transgene *UAS-mMetRS*^*L274G*^*-EGFP*. Flies carrying the wild-type form, that is, *UAS-mMetRS*^*wt*^*-EGFP*, were generated as controls. Furthermore, UAS constructs encoding myc-epitope- or EGFP-tagged *Drosophila* MetRS variants mutated in the same manner at the respective position, that is, Leu^262^ (Supplementary Fig. 3), were used to generate transgenic flies (*UAS-dMetRS*^*L262G*^*-3xmyc, UAS-dMetRS*^*L262G*^*-EGFP*). We then used FUNCAT to assess whether targeted expression of the mutated MetRS variants allows for cell-type-specific labelling of *Drosophila* proteomes. After testing various concentrations (Supplementary Fig. 5, also see Supplementary Note 1), ANL was food supplied at 4 mM throughout this study. As exemplified for dMetRS^L262G^-EGFP in Fig. 1, we found that at larval neuromuscular junctions only cells expressing any of the mutated MetRS variants show incorporation of ANL in either neurons (*elav*^*C155*^*-Gal4* driver; Fig. 1a), glia cells (*repo-Gal4* driver; Fig. 1b) or muscle cells (*C57-Gal4* driver; Fig. 1c). Likewise, ANL-labelled proteins were detectable only in appropriate subregions of wing disc epithelia when *ptc-Gal4* was used to drive expression of the mutated enzyme (Fig. 1d). Expression of wild-type mMetRS-EGFP did not result in detectable labelling (Supplementary Fig. 4a), whereas C57*-Gal4*-driven mMetRS^L274G^-EGFP expression leads to ample ANL incorporation into muscles of larval body walls (Supplementary Fig. 4b). On targeted expression of dMetRS^L262G^-EGFP in muscle cells, TAMRA-tagged ANL-labelled proteins are found throughout the cell (Fig. 1c), that is, in the cytosol, abundantly in the nuclei and in the area of the bouton surrounding subsynaptic reticulum. This implies that ANL-harbouring proteins belong to different categories including soluble and membrane-associated proteins.

### Cell-type-specific bulk labelling of proteins by ANL-BONCAT

**Figure 2:**
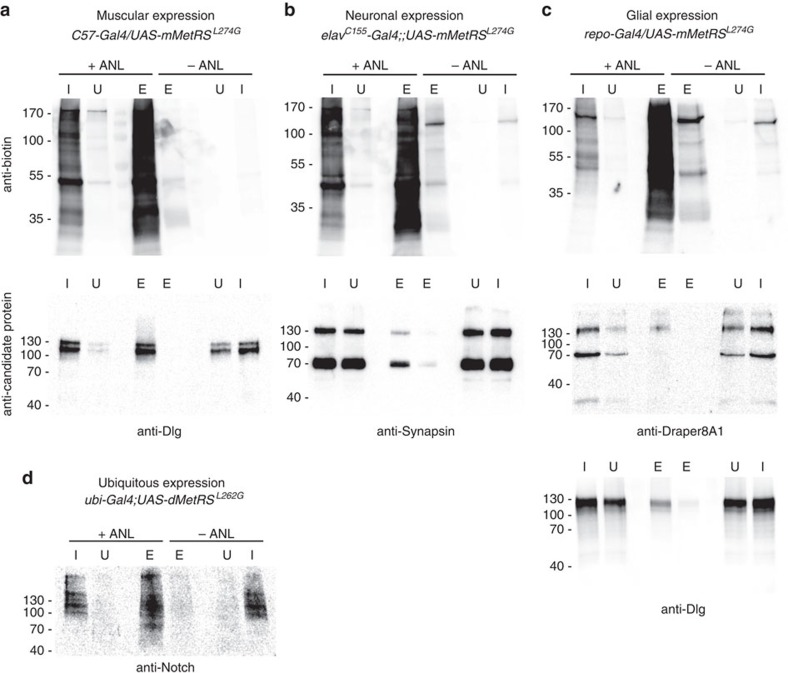
Tagging of ANL-labelled proteins in *Drosophila* larvae and flies. L3 stage larval body walls (**a**) or brains of L3 stage larvae (**d**) were dissected after chronic ANL feeding using 4 mM ANL concentration. Heads from adult *Drosophila* flies (**b**,**c**) were collected 0–3 days post eclosion after chronic ANL feeding using an ANL concentration of 4 mM. ANL is efficiently incorporated into muscle (**a**), neuronal (**b**) or glial proteins (**c**) when *Drosophila* larvae (**a**,**d**) or adult flies (**b**,**c**) express mMetRS^L274G^-EGFP or dMetRS^L262G^-EGFP. ANL-labelled proteins were tagged by a biotin-alkyne affinity-tag, and purified via NeutrAvidin agarose. Tagged input (I, before NeutrAvidin purification), unbound (U, no ANL-containing proteins) and eluted (E, enriched ANL-labelled proteins after NeutrAvidin purification) fractions from ANL labelled and control samples were run in mirror-imaged order on SDS-PAA gels, blotted and probed with anti-biotin antibody. Effective ANL labelling and subsequent biotin tagging were verified for selected marker proteins (‘anti-candidate protein'), that is, Dlg (**a**) in muscles, Synapsin in neurons (**b**) as well as Draper I (**c**) and to a small amount Dlg (**c**) in glia cells. Furthermore, the intracellular domain (**d**) of the transmembrane protein Notch was found to be ANL labelled when dMetRS^L262G^-EGFP is expressed ubiquitously.

Cell-specific labelling by FUNCAT could reflect incorporation of ANL into many different proteins or just into a few abundantly expressed proteins. To discriminate between these possibilities, we analysed cell-type-specific ANL-labelled proteomes by western blot (Fig. 2). Specifically, we expressed mMetRS^L274G^-EGFP in larval muscles, adult neurons or adult glial cells and reared the animals on food with or without ANL. Protein lysates from larval body walls or adult heads (comprising neurons and glia) were subjected to CuAAC reaction with a biotin-alkyne affinity tag, subjected to affinity purification and then analysed by western blot using an anti-biotin antibody. Lysates from ANL-fed flies gave rise to strong signals virtually across the entire molecular weight range. Only a few distinct bands were detectable in control samples, most likely reflecting endogenously biotinylated proteins (Fig. 2a–c; ‘anti-biotin'). This demonstrates that in all three cell types, ANL became efficiently incorporated into at least a broad variety of proteins. We next aimed to assess ANL incorporation into selected proteins. Western blot analysis on larval body wall extracts, which comprise both muscle and epithelial cells, typically reveals two bands when probed with antibodies against the scaffold protein discs large (Dlg), corresponding to isoforms DlgA and DlgS97. On muscle-specific expression of mMetRS^L274G^-EGFP both bands were detected at virtually the same ratio in BONCAT treated, affinity-purified extracts from ANL-fed larvae (Fig. 2a; ‘anti-candidate protein'). This demonstrates that both DlgA and DlgS97 are expressed in muscles as inferred previously from more indirect evidence^33^. We further detected ANL incorporation into the synaptic vesicle protein Synapsin in head lysates from flies with *elav*^*C155*^*-Gal4*-driven mMetRS^L274G^-EGFP (Fig. 2b; ‘anti-candidate protein') and into the glial engulfment receptor Draper in head lysates from flies with *repo-Gal4*-driven mMetRS^L274G^-EGFP (Fig. 2c; ‘anti-candidate protein'). The latter also contained ANL-labelled Dlg (Fig. 2c; ‘anti-candidate protein'), pointing to hitherto largely unnoticed glial expression of Dlg in the adult brain. Control samples from flies that received no ANL or expressed mMetRS^wt^-EGFP yielded no specific bands (Fig. 2). Very similar results were obtained when dMetRS^L262G^-EGFP variants were employed (Supplementary Fig. 5), Thus, cell-type-specific ANL-labelling can be tracked at both the level of bulk protein synthesis as well as at the level of individual proteins.

We performed two independent assays to substantiate that MetRS^LtoG^ replaces protein internal rather than just amino terminal methionine residues by ANL. First, we exploited that processing of the transmembrane protein Notch gives rise to a large cytoplasmic fragment^34^. This fragment was clearly detectable as a smear in ANL-labelled fractions derived from brain lysates of ANL-fed larvae that expressed dMetRS^L262G^-EGFP ubiquitously (*ubi-Gal4*) (Fig. 2d). Given the presence of 32 methionine residues in the Notch intracellular fragment, this smear most likely represents different levels of ANL incorporation and subsequent click chemistry tagging. In addition, we subjected anti-GFP immunoprecipitates from dMetRS^L262G^-EGFP expressing, ANL-fed flies to mass spectrometry and found internal peptides of the enzyme harbouring ANL instead of methionine (Supplementary Fig. 6).

### Limited side effects by chronic ANL incorporation

**Figure 3:**
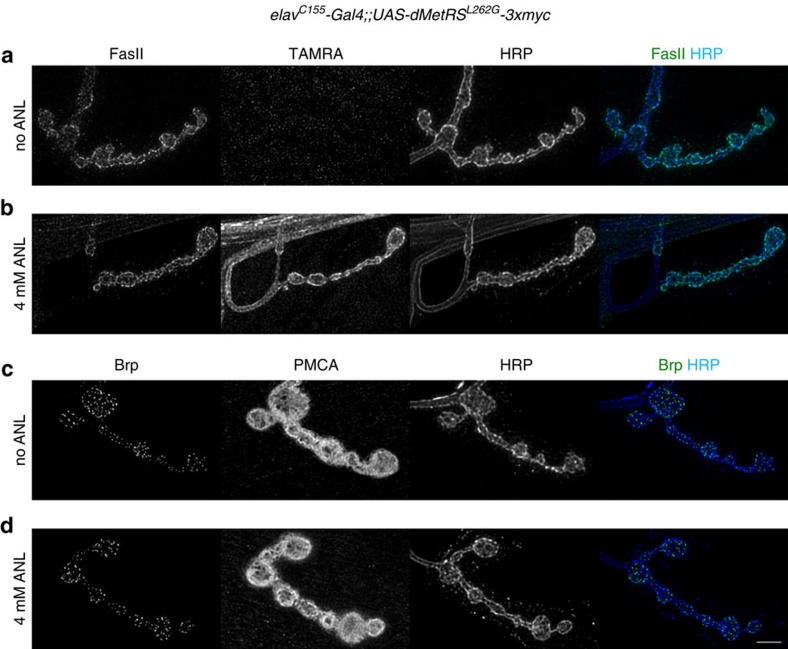
Neuronal ANL incorporation leaves NMJ morphology and prominent NMJ marker proteins unaffected. Motor nerve terminals at NMJs from *elav*^*C155*^*-Gal4;;UAS-dMetRS*^*L262G*^*-3xmyc* L3 larvae fed without (**a**,**c**) or with (**b**,**d**) 4 mM ANL were visualized by the surface marker HRP and assessed for either FUNCAT-mediated TAMRA signals in combination with immunofluorescent labelling of the homophilic cell adhesion molecule FasII (**a**,**b**) or co-stained for the active zone marker Brp and PMCA to visualize the postsynaptic SSR compartment (**c**,**d**). There are no obvious differences between ANL- and non-ANL-reared animals. Scale bar, 5 μm. NMJ, neuromuscular junction; SSR, subsynaptic reticulum.

Having documented MetRS^LtoG^-mediated ANL incorporation into a wide range of proteins, we investigated putative side effects. To this end, we crossed heterozygous *UAS-MetRS*^*LtoG*^*-EGFP* lines to various Gal4 strains and compared MetRS^LtoG^-expressing offspring with their non-expressing siblings (Supplementary Fig. 7). In fact, we noticed that chronic mMetRS^L274G^-EGFP-mediated incorporation of ANL into muscle proteins (*C57-Gal4*) caused an impairment in larval growth (Supplementary Fig. 7a), accompanied by reduced mobility in a larval crawling assay^35^ (Supplementary Fig. 8c) and followed by high lethality during pupal development. Notably, much less pronounced deficits in larval growth and no impairment in larval locomotion were observed when dMetRS^L262G^-EGFP was expressed in the same manner (Supplementary Figs 7a and 8b), despite efficient ANL incorporation (Fig. 1c; Supplementary Fig. 5a–c). Also, depending on which MetRS^LtoG^ variant was used, chronic ANL incorporation into glia cells (*repo-Gal4*) had an effect at 2 mM ANL and at 8 mM ANL (Supplementary Fig. 7a) or no effect (Supplementary Fig. 7a) on the number of eclosed progeny. With one exception (*repo-Gal4/UAS-dMetRS*^*L262G*^*-EGFP*; Supplementary Fig. 7a), we found a correlation between larval growth/eclosion rate and administered ANL concentration (Supplementary Fig. 7a). In case of pan-neuronally (*elav*^*C155*^*-Gal4)* expressed MetRS^LtoG^, however, we repeatedly found that at 2, 4 and 8 mM ANL, the number of eclosed flies was significantly reduced compared with the expected eclosion rate of 1.0 (Supplementary Fig. 7a). Notably, once eclosed survival of adults appeared largely unaffected for both *elav*^*C155*^*-Gal4*- and *repo-Gal4*-driven dMetRS^L262G^-EGFP (Supplementary Fig. 7b). As we did not observe extraordinary pupal lethality in *elav*^*C155*^*-Gal4*-expressing MetRS^LtoG^ variants, we reasoned that MetRS^LtoG^-expressing animals might be aggrieved during larval phases. An inspection of larval neuromuscular junctions by confocal microscopy using common markers did not uncover striking defects (Fig. 3) and, consistently, ANL-reared *elav*^*C155*^*-Gal4;;UAS-MetRS*^*LtoG*^*-EGFP* larvae behaved normally in our crawling assay (Supplementary Fig. 8d,e). Reasoning that rather than a single major effect a variety of subtle side effects of ANL might collectively account for compromised vitality and behaviour, we assayed in addition to the above mentioned larval crawling assay the impact of ANL on adult behaviours and vitality. Focusing on neuronal ANL incorporation (*elav*^*C155*^*-Gal4;;UAS-MetRS*^*LtoG*^*-EGFP*), we used the rapid iterative negative geotaxis assay^35^,^36^, the island assay^37^ and an ethanol sensitivity assay^38^. In general, ANL *per se* did not have effects on any of the behaviours tested in wild-type flies (Supplementary Figs 8a,f, 9a,b, 10a,b and 11a,d). Moreover, in the majority of assays, chronic administration of ANL (that is, throughout the entire development) resulted in no discernible behavioural abnormalities (Supplementary Figs 9c–f and 11b,c). Deficits were only monitored in the negative geotaxis assay for both MetRS^LtoG^ forms (Supplementary Fig. 8g,h) and for mMetRS^L274G^-EGFP-expressing flies in delayed platform clearance in the island assay, whereas the other performances were not affected (Supplementary Fig. 9c,d). Notably, no such deficits were observed when ANL was fed acutely (that is, 48 h; Supplementary Figs 8g,h, 10c–f and 11e,f).

### NCAT labelling correlates with duration of ANL exposure

**Figure 4:**
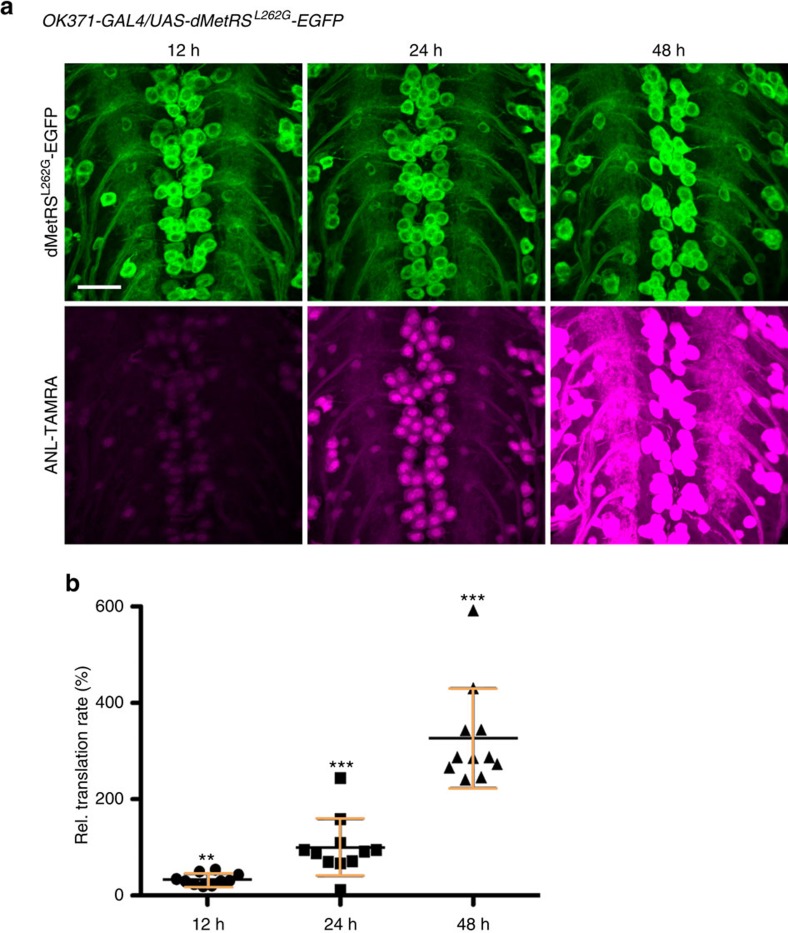
NCAT monitoring of protein synthesis over time. Newly synthesized proteins were monitored by FUNCAT in motor neurons of *OK371-Gal4;UAS-dMetRS*^*L262G*^*-EGFP* third instar larvae that were exposed to ANL for 12, 24 or 48 h (**a**). Selective dMetRS^L262G^-EGFP expression in motor neurons was confirmed by EGFP fluorescence (**a**, upper panel). Fluorescence intensity of TAMRA tagged newly synthesized proteins increased with increasing ANL exposure times (**a**, lower panel). Identical confocal settings were used for acquisition of all images, and representative images are shown. Scale bar, 20 μm. (**b**) Quantification of fluorescence intensities confirmed the positive correlation between FUNCAT labelling intensity and the duration of ANL exposure. Averages±s.d. relative to 24 h ANL exposure (100%) are shown. Mann–Whitney *U*-test, *n*=10–11, **P≤0.01, ***P≤0.001 12 versus 24 h: **P=0.001530673; 12 versus 48 h: ***P=1.08E−04; 24 versus 48 h: ***P=9.34E−05. s.d. (12 h ANL): 11.83; s.d. (24 h ANL): 59.28; s.d. (48 h ANL): 103.50.

**Figure 5:**
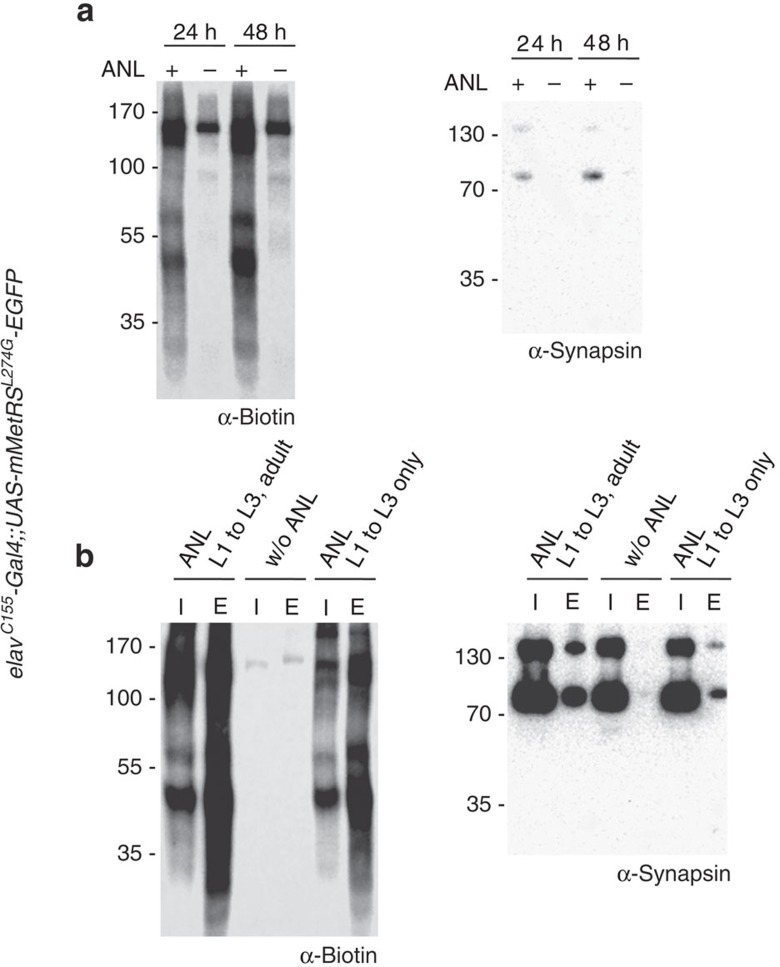
Time course of ANL labelling of neuronal proteins in adult flies. (**a**) Zero to three days old *elav*^*C155*^*-Gal4;;UAS-mMetRS*^*L274G*^*-EGFP* flies were placed either onto ONM with 4 mM ANL or without ANL. After 24 and 48 h, heads from adult *Drosophila* flies were collected and ANL incorporation was addressed by BONCAT. Representative immunoblots showing the extent of ANL-labelled proteins and the neuronal marker protein synapsin after NeutrAvidin purification. The signal intensities for both overall newly synthesized proteins and synapsin increases from 24 to 48 h of ANL incorporation periods. (**b**) The assessment of protein-incorporated ANL into proteins and into synapsin, which is transmitted from larval neurons throughout the pupal phase into adult brain proteins, is rather high compared with flies that were on ANL-containing ONM through all developmental stages. Depicted immunoblots show input (I, before NeutrAvidin purification) and eluted fractions (E, enriched ANL-labelled proteins after NeutrAvidin purification) of samples.+ represents 4 mM ANL and − represents w/o ANL.

To validate the versatility of the method, we tested shorter feeding and labelling periods in larvae and adult flies. Using the *OK371-Gal4* driver line to express dMetRS^L262G^-EGFP in motor neurons, we evaluated FUNCAT signal intensity in the ventral nerve cord after 12, 24 and 48 h of ANL exposure in L3 larval body wall preparations (Fig. 4a,b). After feeding larvae for 12 h with 4 mM ANL, newly synthesized ANL-harbouring proteins were mainly detectable in the nuclei and to a lesser extent in the cytosol of cell bodies of motor neurons (Fig. 4a). After 24 h, bright staining was observable in the nuclei, the somatic cytosol and outgoing processes (Fig. 4a,b). Labelling intensities reached very high levels throughout the neurons after 48 h of exposure (Fig. 4a,b). We observed increasing variation of labelling intensities with increasing ANL incorporation times (Fig. 4b) possibly due to distinct inter-individual translation rates. Next, we performed the short-term labelling experiment in adult flies in conjunction with BONCAT analysis (Fig. 5a). Here we observed the same increase both in global protein synthesis as well as on single protein level for synapsin within adult head lysates from 24 to 48 h of feeding time (Fig. 5a).

During metamorphosis, the *Drosophila* nervous system undergoes a series of drastic changes and reorganization steps (reviewed in ref. 39). We wondered whether ANL labelling would outlast this phase and performed a metabolic pulse-labelling experiment in *elav*^*C155*^*-Gal4;;UAS-mMetRS*^*L274G*^*-EGFP* animals. Larvae were allowed to feed *ad libitum* on ANL-containing food for 4 days. Half of wandering L3 stage *elav*^*C155*^*-Gal4;;UAS-mMetRS*^*L274G*^*-EGFP*-expressing larvae were put onto food without ANL (chase group) until eclosion, whereas their siblings remained on ANL-supplemented food. Figure 5b shows the western blot profile for both the groups. Although there is a clear reduction in ANL-harbouring proteins both on the global level as well as for synapsin (Fig. 5b) in the chase group as compared with the long-term labelling group, a substantial amount of ANL-bearing proteins can be detected in the chase group. It remains elusive, however, whether the remaining signal is derived from ‘surviving' larval proteins or whether ANL was reused, for example, following the apoptosis of neurons.

## Discussion

Small chemical reporters have helped to shed light on the dynamics of a variety of biological molecules. This holds especially true for alkyne- and azide-bearing fatty acids, sugars and non-canonical amino acids, which can be covalently coupled in CuAAC reactions with fluorescent tags for visualization or with tags bearing an affinity moiety for separation and biochemical and mass spectrometry analyses. The non-canonical amino acids AHA and HPG have been used to explore protein und nucleosome turnover^9^,^40^,^41^, to monitor local protein synthesis^17^,^20^ and to identify locally synthesized proteins in neuronal processes^21^,^42^ as well as to perform quantification of proteome dynamics^43^,^44^,^45^,^46^ in different cell culture models and in entire larval zebrafish^22^. Importantly, these NCAT techniques enable no cell-type-specific resolution as all cells *per se* incorporate AHA and HPG. To overcome this limitation, the Tirrell and Schuman groups recently used heterologous expression of a mutant MetRS from *E. coli* carrying a triple amino-acid mutation in the methionine-binding pocket (EcMetRS^NLL^) to permit incorporation of ANL into proteins made in mammalian (HEK293) cells^26^. However, as pointed out by the authors, the EcMetRS^NLL^ mutant enables ANL incorporation only at the very N terminus of proteins as it charges ANL only onto the initiator methionyl-tRNA. As it is thought that about 80%^47^,^48^,^49^ of proteins undergo N-terminal methionine cleavage or excision this constitutes a severe limitation^26^. Moreover, the same paper reports that attempts to generate transgenic worms expressing this mutated form under the control of an inducible cell-specific promoter were not successful. Using the single amino-acid mutation leucine to glycine^23^ at position 274 in the murine MetRS or at position 262 in the *Drosophila* MetRS in the evolutionary well-conserved methionine-binding pocket, we were able to solve this problem^26^. We report here the generation of transgenic flies, which allow targeted expression of MetRS^LtoG^ and, as a consequence, ANL incorporation into proteins of selected cell types in living *Drosophila*. Both the murine MetRS^L274G^ and the *Drosophila* dMetRS^L262G^ enable incorporation of ANL into nascent proteins, which can be visualized using the previously described FUNCAT procedure^17^,^18^ or biochemically analysed on global proteome or single protein level with BONCAT^14^,^15^,^42^. Importantly, FUNCAT and BONCAT confirm that only cells bearing the mutated enzyme incorporate ANL during translation. ANL-harbouring proteins cover the entire molecular weight range. Notably, identification of ANL-modified peptides in the tandem mass spectrometry analysis as well as the successful tagging of type I transmembrane proteins such as Draper, which undergo signal peptide cleavage, and the intracellular Notch fragment confirm the substitution of methionine by ANL at any position within polypeptide chains. ANL incorporation is detectable via FUNCAT as early as 12 h of exposing larvae to ANL-containing food with increasing signal intensities after 24 and 48 h. This finding is mirrored for 24 and 48 h of ANL exposure with BONCAT. Interestingly, metabolic pulse labelling of larval neuronal proteins with ANL using *elav*^*C155*^*-Gal4;;UAS-mMetRS*^*L274G*^*-EGFP*-expressing larvae revealed a substantial amount of ANL-harbouring proteins in flies 3 days post eclosion. Whether these proteins have been synthesized in larval stages and carried over into adult flies (many neurons will persist throughout metamorphosis) or whether these proteins reflect incorporation of recycled ANL from apoptotic cell material has to be clarified in future pulse-chase studies.

Proper protein function and localization are of critical importance for proteomic approaches that aim at characterizing cell-specific proteome dynamics with the ultimate goal to understand physiological and pathological conditions at the level of single cells. Despite the bulk incorporation of ANL at 4 mM, we observed only limited side effects in most of the here applied survival and behavioural assays, in particular when dMetRS^L262G^ was employed. For instance, wings, which allow for a very sensitive readout of patterning defects, remained unaffected when ANL became incorporated into proteins of cells along the anterior–posterior compartment border (Fig. 1d). A reduced number of flies eclosed when ANL was chronically incorporated into neuronal and glial proteomes. In light of future applications, we consider it of pivotal importance that acute incorporation of ANL in adults showed no deficits in any of our assays. It is conceivable, however, that thorough controls for side effects are required according to the specific design of any given experiment. Moreover, our time course experiments showed that shorter times of ANL incorporation into proteins are compatible with detection by both FUNCAT and BONCAT, thus providing a means to minimize side effects even further. Indeed, the possibility to carry out acute pulse labelling by simply feeding ANL expands the range of potential applications tremendously, not at last in behavioural assays related to learning and memory.

With the here presented cell-selective non-canonical amino acid tagging, we addressed the limitations of the previously reported labelling methods with AHA or HPG. Especially, usage of the mutated MetRS (MetRS^LtoG^) does not require a Met-free culture medium or Met-depleted nutrition to incorporate ANL into proteins. It, thus, enables both cell-selective labelling in living animals as well as physiological concentrations of methionine during the labelling procedure.

Using this technique, we investigated protein synthesis rates in a *Drosophila* model for Charcot–Marie–Tooth associated with mutations in glycine-tRNA synthetase (GARS) in an accompanying paper (Niehues *et al.*, 2015). Transgenic flies carrying transgenes for GARS mutants or GARS wild type and dMetRS^L262G^-EGFP were analysed for differences in protein synthesis rates in motor and sensory neurons. ANL-FUNCAT and ANL-BONCAT experiments revealed a significant reduction of protein synthesis rate in larvae expressing GARS mutant isoforms, up to 40–68% of control. In contrast, using AHA was not sensitive enough to detect labelled proteins in genotypes in which translation is inhibited probably due to competition by methionine. Radioactive labelling, in turn, uncovered a similar reduction, which, however, required ubiquitous expression of the mutant GARS proteins to avoid dilution of the effect by non-expressing cells. The use of cell-type-specific metabolic labelling with ANL can resolve such a particular limitation.

We, thus, consider cell-type-specific ANL-FUNCAT and ANL-BONCAT as eligible techniques to study protein synthesis-dependent processes in a complex and behaving organism such as *Drosophila*. While this manuscript was under review, the non-canonical amino acid azidophenylalanine in conjunction with a mutated phenylalanyl-tRNA synthetase was introduced for cell-specific proteome labelling in transgenic worms^50^, further expanding the toolbox of metabolic labelling approaches. In the future, these types of *in vivo* labelling of *de novo* synthesized proteins will facilitate the correlation of molecular changes with processes occurring during synaptic plasticity, or during the course of neurodegenerative events. Furthermore, it will help to unravel the identity of complete secreted proteomes and could be combined with metabolic labelling approaches to tackle post-translational modifications and controlled degradation of proteins as well.

## Methods

### Reagents and antibodies

All reagents were American Chemical Society (ACS) grade and purchased from Sigma unless noted otherwise. AHA was prepared as described^51^. ANL was prepared as described for AHA via copper-catalysed diazo transfer^51^. Briefly, *N*_α_-(*tert*-butoxycarbonyl)-L-lysine was converted to Boc-protected ANL using the azidification reagent triflic azide. The free amino acid was obtained on treatment with acid and was purified by ion-exchange chromatography. Biotin-alkyne tag (biotin-polyethylene oxide (PEO)-propargylamide) was synthesized as described^15^.

For western blots, rabbit polyclonal antibodies against biotin (Bethyl Laboratories Inc.) and GFP (Abcam) were used both at 1:10,000 and mouse monoclonal antibodies (Developmental Studies Hybridoma Bank) against Dlg (4F3), synapsin (3C11), Draper (8A1) and Notch (C17.9C6) were applied at 1:500. Peroxidase-conjugated secondary antibodies (Jackson ImmunoResearch) were used at 1:7,500. For immunofluorescent labelling, mouse monoclonal antibodies (Developmental Studies Hybridoma Bank) against Dlg (4F3), Brp (NC82) and FasII (1F4) were used at 1:500, 1:100 or 1:10, respectively. Rabbit anti-PMCA (plasma membrane calcium ATPase, U.T., unpublished observations) was used at 1:2,000. Dye-conjugated secondary antibodies from goat or donkey (Jackson ImmunoResearch) and goat anti-HRP-Cy5 were used at 1:200 (Jackson ImmunoResearch).

### Transgenic constructs and flies

The coding sequence of mMetRS was PCR amplified from I.M.A.G.E complementary DNA (cDNA) clone 6416029 (GenBank ID BC079643) using 5′-CCG*CTCGAG*GCCACCATGAGACTGTTCGTGAG-3′ as forward primer and 5′-CG*GAATTC*CTTTTTCTTCTTGCCTTTAGGAGTT-3′ as reverse primer. The PCR product was inserted as a XhoI–EcoRI fragment into pEGFP-N2 and the resultant mMetRS^wt^-EGFP fusion was cloned as a XhoI–AflII fragment in between the respective sites of the fly transformation vector pUAST. A PCR strategy was applied to the original cDNA to generate mMetRS^L274G^. The above primers were used in PCRs with the reverse primer 5′-TCACCAGTGCC*GGC*CCCTATGTCA-3′ or the antiparallel primer 5′-TGACATAGGGGC*CGG*CACTGGTGA-3′, respectively, both covering the desired mutation. The overlapping PCR fragments were annealed to each other and thereupon used as a template to PCR amplify the coding sequence for mMetRS^L274G^ using the outer forward and reverse primers. Subsequent cloning steps were carried out as for mMetRS^wt^. To generate dMetRS^L262G^, we first introduced the respective mutation into an exon fragment amplified from the dMetRS locus (CG15100) of a wild-type fly according to the two-step PCR strategy described above. Specifically, the mutating primers 5′-CATCACCTCGGCG*GGT*CCCTATGTCAAC-3′ and 5′-GTTGACATAGGG*ACC*CGCCGAGGTGATG-3′ were used with outer primers 5′-GGAAGTTCTTGCCGAACAGCCAC-3′ or 5′-CAAAGCCAATGCCGAACCAGCG-3′, respectively. A 375-bp BsmBI–ScaI subfragment of this PCR product was used to substitute the corresponding part in the dMetRS cDNA clone AT05114 (*Drosophila* genomics resource center; GenBank ID AY119445), thereby introducing the mutation and repairing an unwanted missense mutation (A232T). We also noticed that the cDNA lacked 30 bp encoding His^820^ to Glu^829^ (not related to canonical splicing) and therefore used a PCR-derived 215-bp BstEII genomic fragment to replace the respective fragment in the cDNA. However, both the corrected and the Δ30 bp versions of dMetRS^L262G^ were later found to efficiently incorporate ANL. To allow C-terminal fusions with EGFP or a 3 × myc-epitope, the stop codon was removed in a PCR reaction on the cDNA using primers 5′-GTTGATAAACCGAGAGCTGCGTG-3′ and 5′-TGTCGACTTCTTCTTTTTGCCCTTG-3′. The SalI site in the latter primer served for in-frame cloning into pEGFP-N1 in a three-fragment ligation involving the vector cut with EcoRI and SalI, an EcoRI–NcoI fragment from the L262G-mutated cDNA and the NcoI–SalI fragment from the latter PCR product. To obtain the epitope-tagged version, the EGFP sequence was replaced by a 3xmyc fragment flanked by SalI and NotI sites, derived from a PCR on a 3xmyc-tagged *dlg* construct (U.T., unpublished observations). The fusion genes were eventually cloned as EcoRI–NotI fragments into pUAST. Following germline injections at BestGene Inc. (CA, USA) individual lines were established for each construct. On testing various lines, the following MetRS effector strains were used throughout this study: *UAS-mMetRS*^*wt*^*EGFP/(CyO)* (line 6202-1), *UAS-mMetRS*^*L274G*^*-EGFP/(TM6b,Tb Hu)* (line 6202-2), *UAS-dMetRS*^*L262G*^*-EGFP/(TM6b,Tb Hu)* (line 2.1), *UAS-dMetRS*^*L262G*^*-3xmyc/(CyO)* (line 3.4).

### Flies

Unless stated otherwise stocks and crosses were grown at 25 °C in a 14 h/10 h dark–light cycle on Otto normal medium (ONM) containing Agar–Agar (0.83% w/v), mashed raisins (4% w/v), yeast (6% w/v), semolina (5% w/v), sugar beet syrup (2.6% w/v), honey (2.6% w/v) and Nipagin (0.13% (v/v). Dominant markers associated with balancer chromosomes, *CyO, CyO*^*act-GFP*^ and *TM6b, Tb Hu*, were used to establish transgenic and recombinant lines and to identify genotypes of progeny at larval and adult stages in crosses of heterozygous flies. All Gal4 activator strains have been described as folllowing: *elav*^*C155*^*-Gal4* (pan-neuronal^52^)*, repo-Gal4* (glia-specific^53^), *OK371-Gal4* (motoneuronal^54^), *C57-Gal4* (muscle specific^55^), *ptc*^*559.1*^-Gal4 (subregional in imaginal discs^56^) and *ubi-Gal4* (all cell types, obtained from Bloomington stock center).

### Metabolic labelling of proteins with ANL

AHA was supplied at 4 mM, whereas ANL was supplied at 2, 4 or 8 mM (as indicated) in the food. For long-term labelling, crosses were continuously reared on AHA- or ANL-containing medium and offspring analysed at larval stage L3 or as adults. To assess shorter labelling time windows in adult flies, crosses were reared on ONM and adult offspring were transferred onto 4 mM ANL-containing ONM 0 to 3 days post eclosion and analysed for ANL incorporation after 24 and 48 h. To investigate short-term metabolic labelling in larvae, 2-h egg collections were performed and larvae were raised on Jazz-Mix *Drosophila* food (Fisher Scientific) at 25 °C. Three groups were defined that were first raised on normal food and then transferred to 4 mM ANL-containing food; group A: 72 h w/o ANL/48 h with ANL; group B: 96 h w/o ANL/24 h with ANL; and group C: 108 h w/o ANL/12 h with ANL.

### Toxicity test of ANL to *Drosophila* larvae and flies

To test any putative toxicity of ANL to *Drosophila* larvae, the body weight of wandering L3 stage larvae was determined using an ultra fine scale (Sartorius). Body weights of either dMetRS^L262G^-EGFP- or mMetRS^L274G^-EGFP-expressing larvae were compared between larvae reared on ONM with 2, 4 and 8 mM ANL or without ANL. Data were analysed using one-way analysis of variance (ANOVA) with Dunnett *post hoc* tests. To test for a linear relationship between ANL incorporation and larval body weight, linear regression analyses were performed. To analyse any putative toxicity of ANL to adult *Drosophila* flies, five virgin female flies of the driver strain (either *elav*^*C155*^*-Gal4* or *repo-Gal4*) were crossed with five male flies of the UAS strain (either *UAS-dMetRS*^*L262G*^*-EGFP* or *UAS-mMetRS*^*L274G*^*-EGFP*) and reared on ONM with 2, 4 and 8 mM ANL or without ANL. Parental flies were removed from the offspring before reaching the pupal stage. After hatching of the first offspring generation flies (∼10–11 days after the crossing was started), the number of offspring was counted every second day and compared between the different groups expressing either dMetRS^L262G^-EGFP or mMetRS^L274G^-EGFP versus flies not expressing dMetRS^L262G^-EGFP or mMetRS^L274G^-EGFP. To determine the metamorphosis and eclosion rate of ANL-incorporating flies, we compared the group size relative with the segregation after Mendel's law (expressing either dMetRS^L262G^-EGFP or mMetRS^L274G^-EGFP) reared either on ONM with 2, 4 and 8 mM ANL or without ANL. Statistical analyses were performed using one sample *t*-tests against a theoretical mean of 1.0. To investigate the correlation between ANL incorporation and eclosion rate, linear regression analysis with a 95% confidence interval was performed.

Investigation of the survival rate of MetRS^LtoG^-EGFP-expressing flies was performed by crossing virgin female flies of different driver strains (*elav*^*C155*^*-Gal4* or *repo-Gal4*) to male flies of the UAS strain (either *UAS-dMetRS*^*L262G*^*-EGFP*, *UAS-mMetRS*^*L274G*^*-EGFP*), rearing them either on ONM with 2, 4 or 8 mM, or on ONM without ANL. At 0–3 days post eclosion, 10 flies each (five female and five male) were transferred either to ONM with the same as previous ANL concentration (for example, 2 mM ANL—2 mM ANL) or to ONM without ANL (for example, 2 mM ANL—w/o ANL). Over a total period of 14 days, the number of living flies was determined every other day.

### Click chemistry (CuAAC) and detection of tagged proteins

*Visualization of ANL- or AHA-labelled proteins using FUNCAT*. Larval body walls were dissected in haemolymph-like 3 (HL-3) solution (70 mM NaCl, 5 mM KCl, 20 mM MgCl_2_, 10 mM NaHCO_3_, 115 mM sucrose, 5 mM trehalose, 5 mM HEPES pH 7.2) with 0.1 mM Ca^2+^ and pre-fixed with two to three drops of 4% paraformaldehyde (in 1 × PB (phosphate buffer, 0.1 M Na_2_HPO_4_, 0.1 M NaH_2_PO_4_) pH 7.2) for 1 min. After exchanging the solution to 4% paraformaldehyde in PB pH 7.2 body walls were incubated for 30 min at room temperature under gentle agitation. Following fixation, body walls were washed three times with PBT (phosphate buffer red saline pH 7.2+0.2% v/v Triton X-100) and another three times with 1 × PBS pH 7.8 (137 mM NaCl, 2.7 mM KCl, 4.3 mM Na_2_HPO_4_, 1.4 mM KH_2_PO_4_) both for 15 min each at room temperature under gentle agitation. ANL-labelled proteins were tagged by mixing triazole ligand (TBTA, Sigma-Aldrich; 200 mM in dimethylsulfoxide; 1:1,000), TAMRA-alkyne tag (Invitrogen; 1 mM; 1:5,000) or Atto647N alkyne tag (ATTO-TEC GmbH; 1 mM; 1:5,000), tris(2-carboxyethyl)phosphine solution (Sigma-Aldrich; 400 mM; 1:1,000) and CuSO_4_ solution (Sigma-Aldrich; 200 mM; 1:1,000) in 1 × PBS pH 7.8. After each addition, the solution was mixed thoroughly for 10 s using a high-speed vortexer. Body walls were incubated with 200 μl of this mixture overnight at 4 °C under gentle agitation. Subsequently, body walls were washed three times with 1 × PBS–Tween (PBS pH 7.4+1% v/v Tween-20) and PBT for 15 min each at room temperature under gentle agitation before incubation with primary and secondary antibodies. Motor nerve terminals were stained with a primary anti-HRP-Cy5 antibody (1:200 in PBT) for 1 h at room temperature. After immunostaining, body walls were washed once more three times for 15 min each with PBT at room temperature under gentle agitation. Finally, body walls were mounted in VectaShield (Vector Laboratories) and stored at 4 °C until imaging.

For analysis of short-term labelling by FUNCAT, larvae were reared on Jazz-Mix *Drosophila* medium as described in the section Metabolic labelling of proteins with ANL. Fixation and FUNCAT reaction were carried out as described above.

*Identification of AHA- and ANL-labelled proteins using BONCAT*. Protein extraction was performed either from larval body walls (final amount of 2.5–3.5 mg), from 60 larval brains, or from 20 heads of adult *Drosophila* flies. Body walls or larval brains were dissected in HL-3 solution with 0.1 mM Ca^2+^. *Drosophila* heads were collected after anaesthetizing flies with CO_2_ by cropping heads from the body. Body walls, larval brains or fly heads were transferred to glass homogenizers (Wheaton). Protein extraction was carried out by homogenizing body walls, larval brains or fly heads in 100 μl 0.5% SDS in 1 × PBS pH 7.8–2 × protease inhibitor (PI, Roche) w/o EDTA+Benzonase (Sigma, ≥250 units per μl) per 2.5–3.5 mg of body wall material, per 60 larval brains or per 20 fly heads. Homogenates were incubated for 20 min at room temperature under permanent agitation before boiling at 95 °C for 7 min. After cooling down on ice, 20% (v/v) Triton X-100 (Sigma) was added to a final concentration of 1% (v/v), and homogenates were diluted with 1 × PBS, pH 7.8, 2 × PI w/o EDTA to a final concentration of 0.1% (w/v) SDS and 0.2% (v/v) Triton X-100. After an incubation for 1 h at 4 °C under permanent agitation, lysates were spun down at 3,000*g*, 5 min, 4 °C. The resulting supernatants (‘lysates') were transferred to Eppendorf tubes and ANL- or AHA-labelled proteins were tagged by adding triazol ligand (Sigma-Aldrich, 200 mM in dimethylsulfoxide; 1:1,000), biotin-PEO_3_-alkyne tag (25 mM; 1:1,000) and copper(I)bromid suspension (Sigma-Aldrich, 10 mg ml^−1^; 1:100). After each addition, the solution was mixed thoroughly for 10 s using a high-speed vortexer. The samples were incubated overnight at 4 °C under permanent agitation. Subsequently, samples were spun down at 3,000*g* for 5 min at 4 °C. The supernatants were transferred onto ZEBA desalting spin columns (Thermo Scientific) to remove excess CuAAC reagents. Desalting was carried out according to the manufacturer's instructions. Protein concentrations between samples were adjusted to the same concentration via amidoblack assay. ANL-labelled proteins were purified using high-capacity NeutrAvidin agarose (Thermo Scientific). For this, a defined volume of high-capacity NeutrAvidin agarose suspension was equilibrated with three washes of 1 ml 1% (v/v) Igepal in 1 × PBS, pH 7.8 each, inverting the tube several times and a subsequent centrifugation step (3,000*g*, 5 min, 4 °C). Tagged lysates were incubated with 1% Igepal for at least 20 min at 4 °C under permanent agitation. Lysates were then incubated with NeutrAvidin agarose overnight at 4 °C under permanent agitation. Afterwards, the supernatants were transferred to new Eppendorf tubes. NeutrAvidin agarose was washed five times for 5 min each with 1% (v/v) Igepal in 1 × PBS, pH 7.8 and three times for 5 min each with 1 × PBS, pH 7.8 as described above. ANL-labelled biotin-tagged proteins were eluted with 2 × SDS sample buffer (0.5 volume of the suspension volume) for 7 min at 95 °C. Eluates were collected after a centrifugation step, transferred to new tubes and processed for western and dot blot analysis as described^15^. Uncropped scans of crucial western blots are included as Supplementary Fig. 12.

### Microscopy and statistical analyses

Images of larval body wall muscles, central nervous system and imaginal discs were acquired on a Leica-SP5 confocal microscope. Images of time course experiment were acquired using an AxioObserver.Z1 microscope equipped with a LSM 700 confocal unit (Carl Zeiss AG, Oberkochen, Germany). Original confocal data and images were processed using the ZEN2010 software (Carl Zeiss AG, Oberkochen, Germany) and the FigureJ plugin for FIJI software^57^ (Laboratory for Optical and Computational Instrumentation, Madison, USA). Identical confocal settings were used for all comparative analyses including image acquisition of motor neuron cell clusters for FUNCAT time course experiments. Fluorescence intensities were quantified using FIJI software^57^. For quantification, mean intensities of all cells within one cluster from each central nervous system were taken as a single biological replicate (*n*=10–11). For statistical analysis, Mann–Whitney *U*-test was used, because the data were not normally distributed. *P≤0.05, **P≤0.01, ***P≤0.001; 12 versus 24 h: *P*=0.001530673=**; 12 versus 48 h: *P*=1.08E−04=***; 24 versus 48 h: ***P=9.34E−05.

### Purification of EGFP-tagged dMetRS^L262G^ for MS analysis

Body walls of 16 *C57-Gal4/ UAS-dMetRS*^*L262G*^*-EGFP*, ANL-fed (ONM with 4 mM ANL) third instar larvae were dissected in ice-cold HL-3 solution as described under section Click chemistry (CuAAC) and detection of tagged proteins. The μMACS isolation kit (Mylteni Biotec) was used in combination with μMACS anti-GFP microbeads according to the manufacturer's instructions. Briefly, proteins were isolated using 400 μl lysis buffer (2 × PI w/o extra EDTA) provided in the kit and mixed with 80 μl μMACS anti-GFP beads. Following incubation on ice for 1 h the suspension was loaded onto a μMACS column, pre-equilibrated with 200 μl lysis buffer. The flow through was collected as a control. After five washes EGFP-tagged proteins were eluted in two elution steps with subsequent pooling of the two eluate fractions using the elution buffer recommended in the manufacturer's protocol but without EDTA. Eluted fractions (100 μl) were separated on 10% sodium dodecylsulfate polyacrylamide (SDS-PAA) gels (1.5 mm) and silver stained according to a modified protocol^58^. Corresponding bands at 130 kDa were cut out and destained using 50 mM sodiumthiosulfatepentahydrate (Na_2_S_2_O_5_ 5H_2_O, AppliChem) and 15 mM potassium cyanoferrate (C_6_FeK_3_N_6_, Sigma).

Proteins in the gel pieces were reduced with 2.8 mM dithiothreitol (60 °C for 30 min), modified with 8.8 mM iodoacetamide in 100 mM ammonium bicarbonate (in the dark, room temperature for 30 min) and digested in 10% acetonitrile and 10 mM ammonium bicabonate with modified trypsin (Promega) overnight at 37 °C. A second digestion was carried out for 4 h.

### Mass spectrometry analysis

The peptides were resolved by reverse phase chromatography on 0.075 × 200-mm fused silica capillaries (J&W) packed with Reprosil reversed phase material (Dr Maisch GmbH, Germany). The peptides were eluted with linear 94-min gradients of 5–28% and 12 min at 95% acetonitrile with 0.1% formic acid in water at flow rates of 0.15 μl min^−1^. Mass spectrometry was performed by Q-Exactive plus (Thermo) mass spectrometer in a positive mode using repetitively full MS scan followed by collision induces dissociation (HCD) of the 10 most dominant ions selected from the first MS scan.

The mass spectrometry data were analysed by the Proteome Discoverer software version 1.4 using the Sequest search engine versus the *Drosophila* section of the Uniprot database with 1% false discovery rate and specific database containing the dMetRS^L262G^-EGFP sequence.

### Behavioural assays

Virgin driver line females (30–100; *elav*^*C155*^*-Gal4, C57-Gal4*) were crossed with half the number of heterozygous *UAS-MetRS*^*LtoG*^*-EGFP* line males and raised on ONM or 4 mM ANL-containing ONM (chronic ANL feeding). The number of males and females per cross was the same for groups that were compared. After 3–6 days, the parental generation was transferred onto fresh ONM with or without 4 mM ANL. Another 4–6 days later the parental generation was disposed. Zero to four days (chronic) or 0–3 days (acute) post eclosion the progeny of a cross was CO_2_ anaesthetized and the *UAS-MetRS*^*LtoG*^*-EGFP*-positive flies or wild-type flies were sexed, collected in groups of 10 flies per vial, and placed onto fresh ONM or 4 mM ANL-containing ONM. In chronic or acute ANL-feeding experiments, flies were tested 18- or 48-h later, respectively.

For larval behavioural analyses after acute ANL feeding, crosses were reared on ONM for 1–2 days and transferred onto fresh ONM for 4–6 h. After 72±2 h at 25 °C, the larvae were washed out of the food with warm tap water and rinsed into a mesh basket before being transferred onto ONM or 4 mM ANL-containing ONM with a brush. Another 24 h later, the larvae were tested in the larval crawling assay. We adopted our protocol for the larval crawling assay from ref. 35. Ten *UAS-MetRS*^*LtoG*^*-EGFP*-expressing third instar larvae of a certain group were centred on a 2% agarose (Biozym) containing Petri dish over 0.2 cm^2^ grid graph paper and videotaped. The number of grid lines each larva crossed within 1 min was determined and a mean was taken from the values for the 10 larvae. The progeny of at least three independent crosses was analysed. Student's *t*-tests were used to test for statistical differences in crawling behaviour between larvae raised on ONM and larvae raised on 4 mM ANL-containing ONM.

We adopted our protocol for rapid iterative negative geotaxis assay from refs 35. A climbing apparatus was generated by placing an empty polystyrene vial (25 × 95 mm, VWR) with a circle mark at 8 cm from the bottom onto the fly-containing food vial. The two vials were fixed with tape. The flies were allowed to acclimatize to this setting for >5 min. The number of flies that passed the 8-cm mark within 10 s after being tapped to the bottom of the vial was recorded. This procedure was repeated 10 times with an inter-trial interval of 3 min and a mean was taken from these measurements (counted as one replicate) for each group. The progeny of at least three independent crosses was analysed. Student's *t*-tests were used to test for statistical differences in climbing behaviour between flies raised on ONM and flies fed on 4 mM ANL-containing ONM. There were no differences between male and female performances. Therefore, the data were pooled.

We adopted our protocol for the island assay from ref. 37. Flies were videotaped while released on a 12.7 × 8.5 cm^2^ platform (island) in the middle of a watery soap bath (31.2 × 15 cm^2^). We analysed the percentage of flies jumping, running, sitting, or flying after being released on the platform (Student's *t*-tests) and determined the time needed to clear the platform. Differences in the time to clear the platform between flies reared on ONM or on 4 mM ANL-containing ONM were analysed with repeated measurement ANOVAs. There were no differences between male and female performances. Therefore, the data was pooled.

We adopted our protocol for ethanol sensitivity from ref. 38. Vials (25 × 95 mm, VWR) containing 10 flies were sealed with a 1 ml 97% ethanol (Fisher, stained with commercially available blue food dye) moistened plugs. The flies were videotaped for 20 min. The number of mobile flies was determined every minute. We analysed resistance to ethanol intoxication by comparing the number of mobile flies over the time course of 20 min (repeated measurement ANOVAs) and by comparing half-maximal sedation (ST50, Student's *t*-tests) of ANL-treated and untreated flies. There were no differences between male and female performances. Therefore, the data were pooled.

## Additional information

**How to cite this article:** Erdmann, I. *et al.* Cell-selective labelling of proteomes in *Drosophila melanogaster*. *Nat. Commun.* 6:7521 doi: 10.1038/ncomms8521 (2015).

## Supplementary Material

Supplementary Information Supplementary Figures 1-12, Supplementary Table 1-4, Supplementary Note 1 and Supplementary Reference
