# Supplementary material for: Cell-selective labelling of proteomes in Drosophila melanogaster
Source: Nat Commun. 2015 Jul 3;6:7521. doi: 10.1038/ncomms8521 (PMC4507001; doi:10.1038/ncomms8521)
Supplement: Supplementary Information — Supplementary Figures 1-12, Supplementary Table 1-4, Supplementary Note 1 and Supplementary Reference [file ncomms8521-s1.pdf]

Supplementary Figure 1:

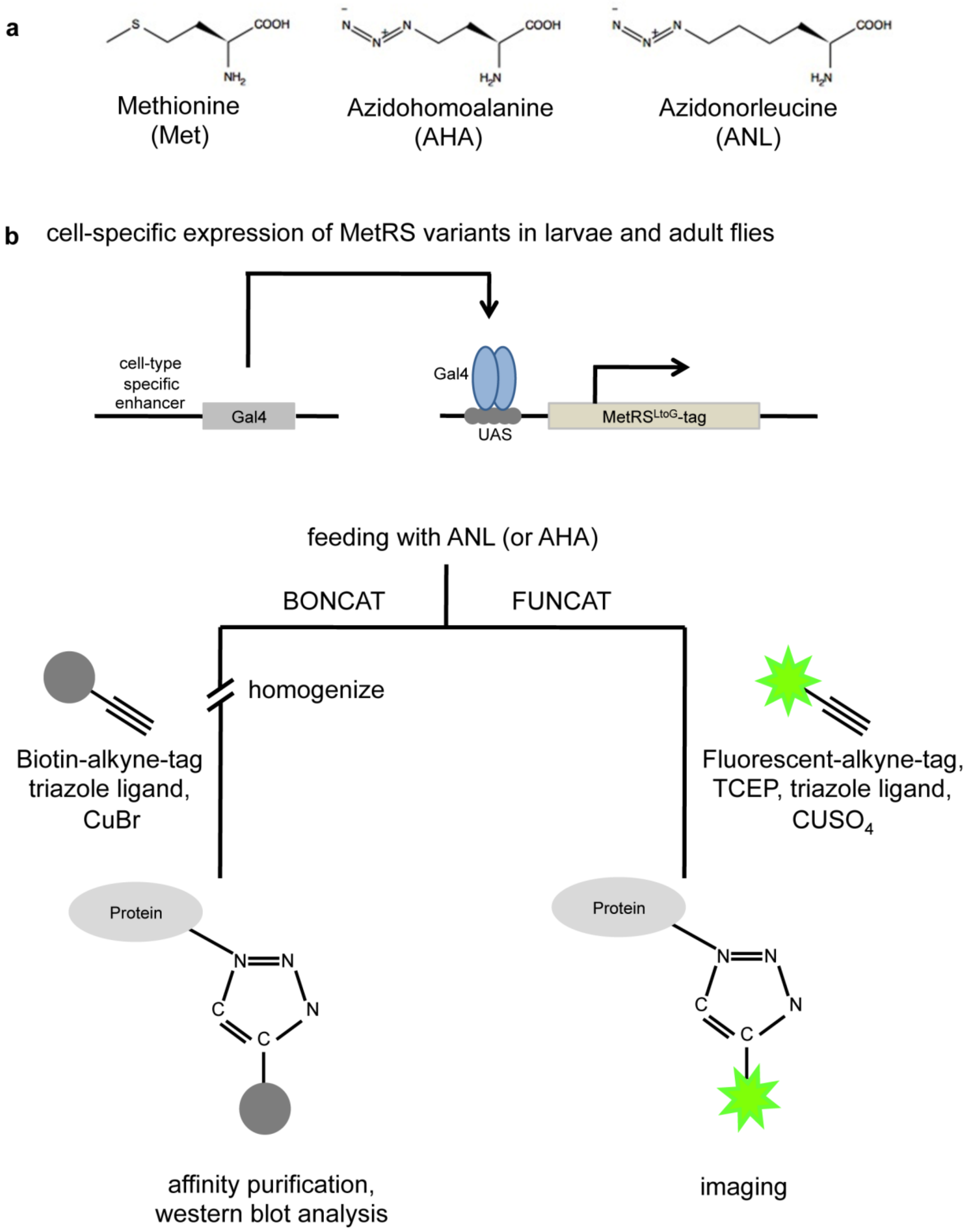

AHA- (ubiquitious) and ANL-labeling (cell type-specific) of *Drosophila* proteins and their detection by FUNCAT and BONCAT. (*a*) Methionine (Met) and its surrogates azidohomoalanine (AHA), and azidonorleucine (ANL). (*b*) Gal4-induced expression of EGFP-tagged mMetRS^{L274G} or dMetRS^{L262G} allows for cell type-specific charging of ANL onto methionine–tRNA (MetRS), and its subsequent incorporation

into proteins. AHA is incorporated into proteins of all cells employing the endogenous MetRS. CuAAC using an affinity probe (biotin-alkyne tag) or a fluorescent probe (TAMRA-alkyne tag) mark AHA- or ANL-harboring proteins for biochemical analysis or *in situ* visualization, respectively.

Supplementary Figure 2:

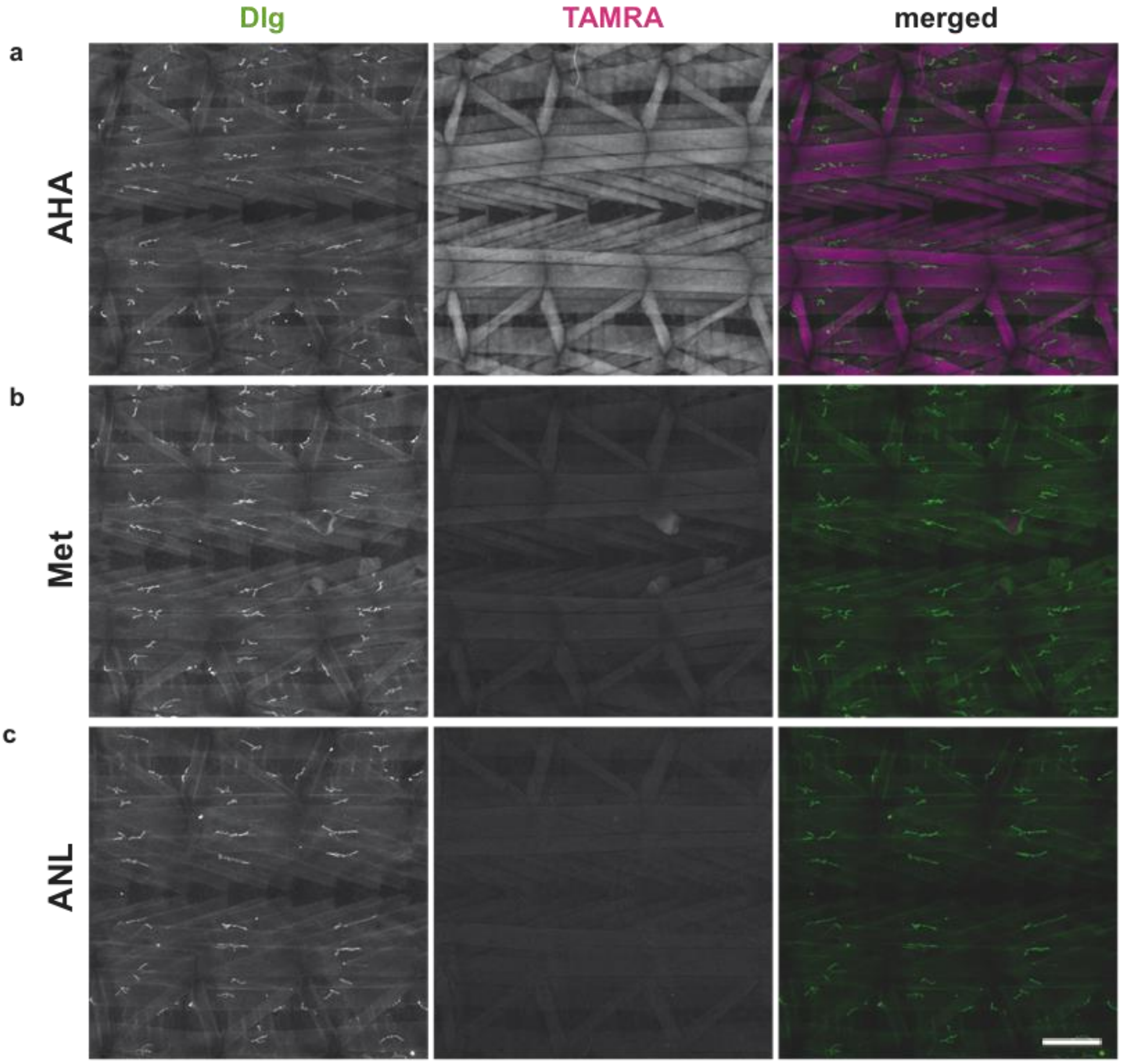

AHA-selectivity and principal specificity of FUNCAT in wild type *Drosophila* larvae. Body wall preparations (abdominal segments A3-A5) from wild type larvae fed with 4 mM AHA (*a*), 4 mM methionine (*b*) or 4 mM ANL (*c*), immunofluorescently labeled for the scaffold protein Dlg. FUNCAT with the red-fluorescent dye TAMRA reveals efficient AHA-incorporation (*a*) whereas no labeling above background levels is evident when ANL was fed (*c*). Expression and NMJ localization of Dlg is not compromised by the incorporation of AHA. Scale bar: 250 µm.

Supplementary Figure 3:

```
                                                                       ↓
E. coli MetRS       --------------------------MTQVAKKILVTCALPYANGSIHLGHMLEH-IQAD   33
Drosophila MetRS    LADIKKSFEASLPG-YVEKKDPKSILPQPGKRNILITAALPYVNNVPHLGNIIGCVLSAD  282
Mus musculus MetRS  ISAAKATFTY---TAPKEIKEERTVLPKPGERNVLITSALPYVNNVPHLGNIIGCVLSAD  294
Danio rerio MetRS   IVTAVAAWEKGLESLPPLKLQQHPVLPVPGERNVLITSALPYVNNVPHLGNIIGCVLSAD  290
C. elegans MetRS    IEEAAQVYSEGLKDFTVVTERKHPVLPQEGKRNVLITSALPYVNNVPHLGNIIGCVLSAD  286
                                                    ::*:*.****.*   ***.::   :.**

E. coli MetRS       VWVRYQRMRGHEVNFICADDAHGTPIMLKAQQLGITPEQMIGEMSQEHQTDFAGFNISYD   93
Drosophila MetRS    VFARYCNLRGHQTFYVGGTDEYGTATETKALQEGCTPRELCDKYHAIHKGIYEWFGIDFS  342
Mus musculus MetRS  IYARYSRSAGYNTLLICGTDEYGTATENKALAENLTPREICDKYFELHNAIYRWFGIGFD  354
Danio rerio MetRS   VFARYCRLRQWNTLYLCGTDEYGTATETKAMEEGLTPREICDKYHAIHADIYRWFGISFD  350
C. elegans MetRS    VFARYGRLRGWNLLYICGTDEYGTATENKAREEGLTPQQICDKYHCIHASIYQWFQIDFD  346
                    ::.** .    :   : . * :**    **     **.::  :    *   :  * * :.

E. coli MetRS       NYHSTHSEENRQLSELIYSRLKENGFIKNRTISQLYDPEKGMFLPDRFVKGTCPK--CKS  151
Drosophila MetRS    HFGRTTTDHQTEICQDMFLKLHKNGYTSSQSVDQLYCNQCEKFLADRFVTGTCPM--CAY  400
Mus musculus MetRS  YFGRTTTQEQTDIVQEAFKDVLKAGYIITESVEQLLCQKCDRFLADRFVEGTCPHPGCGY  414
Danio rerio MetRS   TFGRTTTPQQTKITQDIFQRLLTRGFVLRDTVEQLRCERCARFLADRFVEGVCPF--CGY  408
C. elegans MetRS    FFGRTTTQHQTEIAQDIFWRLHERGFLLEDTVEQLRCEGCQRFLADRFVEGECPH--CRF  404
                     :  * : .: .: :  :  :   *:    ::.**       ** **** * **   *

E. coli MetRS       PDQYGDNCEVCGATYSPTELIEPKSVVSGATPVMRDSEHFFFDLPSFSEMLQAWTRSGAL  211
Drosophila MetRS    DDARGDQCDGCGKLINAVDLKDAKCHMCKATPEVKQSTHIFLSLDKLQQKTTEHLDRELA  460
Mus musculus MetRS  EDARGDQCDKCGKLVNATELIRPRCKVCNSAPVLRSSDQLFIDLPKAEPQLKEWVDKS--  472
Danio rerio MetRS   EEARGDQCDRCGKLINAIELKKPQCKICRSCPVVRSSQHLFLDLPKLEKRLEDWLGKTVP  468
C. elegans MetRS    PEARGDQCDKCGRLINAVELKNPQCKVCKETPVIRSSKHLFLNLPKLEQDLEQWLQTSTA  464
                     :  **:*: **   .  :*   :. :.   * ::.* ::*:.* . .

E. coli MetRS       ------QEQVANKMQEWFESGLQQWDISRDAPYFGFE--IPNAPGKYFYVWLDAPIGYMG  263
Drosophila MRS      KEDNRWSSNAVGITKAWMKLGLDPRCITRDLKW-GTAVPLDGFEKKVFYVWFDAPIGYLS  519
Mus musculus MetRS  --EGGWTHNAKVITRAWLKEGLKPRCITRDLKW-GIPVPHEGFEKKVFYVWFDAPFGYVS  529
Danio rerio MetRS   --GSDWTPNARFIIRSWLRDGLKPRCITRDLKW-GTPVPLEGFEDKVFYVWFDATIGYVS  525
C. elegans MetRS    --AGDWTTNARHITRSWLRDGLKPRCITRDLKW-GTPVPHPDYKEKVFYVWFDAPIGYLS  521
                            :.    : *:. **.   *:**  : *          * ****:** :**:.

E. coli MetRS       SFKNLCDKRGDSVSFDEYWKKD--STAELYHFIGKDIVYFHSLFWPAMLEGS--NFRKPS  319
Drosophila MetRS    ITKCVLG-----DNWTKWWKNP--ENVELFNFVGKDNVAFHAVMFPCSQLGANDNYTVVN  572
Mus musculus MetRS  MTKR-YT-----KEYQQWWQPAKGTDVELFQFMAKDNVPFHSVVWPSVLLAINKGHTLVS  583
Danio rerio MetRS   ITAN-YT-----DQWEKWWKNP--EQVDLYQFMAKDNVPFHGLVFPCSVLGAEDNYTLVK  577
C. elegans MetRS    ITAN-YT-----DQWERWWKNP--QQVELYNFMAKDNVPFHSVVFPCSLLGAQDNYTLVN  573
                                 .: .:*:      .:*:.*:.** * **.:.:*.   .    .   .
```

Sequence alignment of orthologous MetRS. CLUSTAL O (1.2.0) multiple sequence alignment with *E. coli* MetRS (K02671), *Drosophila* MetRS (CG15100), murine MetRS (BC079643), *Danio rerio* MetRS (AAH79643.1), and *C. elegans* MetRS (NP_956370.1). Residues conserved and critical for the binding pocket (according to ref. [1]) are boxed. The arrow indicates the position of the leucine mutated to glycine in dMetRS and mMetRS. Amino acid positions within the respective sequence are indicated on the right.

Supplementary Figure 4:

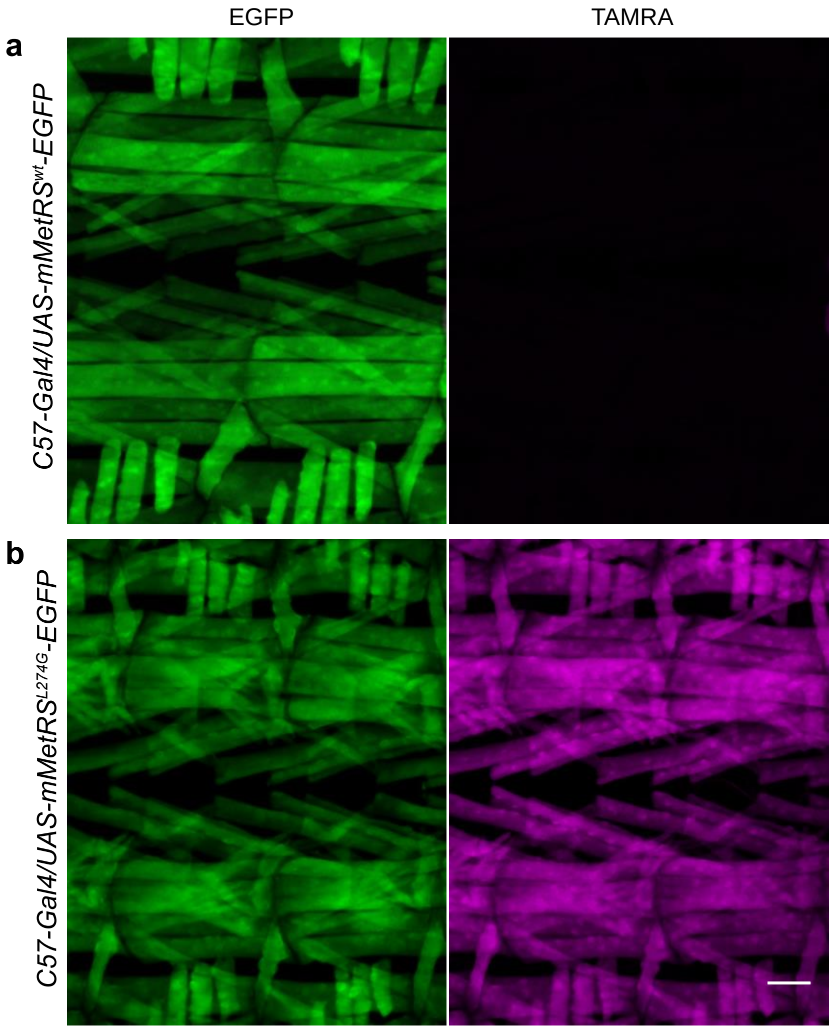

MetRS^{LtoG}-dependent ANL-incorporation. ANL-incorporation into proteins of body wall muscles monitored via FUNCAT upon targeted expression of either wild type mMetRS^{wt}-EGFP (*a*) or mMetRS^{L274G}-EGFP (*b*). TAMRA-staining is restricted to mMetRS^{L274G}-EGFP expressing muscle cells. Scale bar: 100 µm.

Supplementary Figure 5:

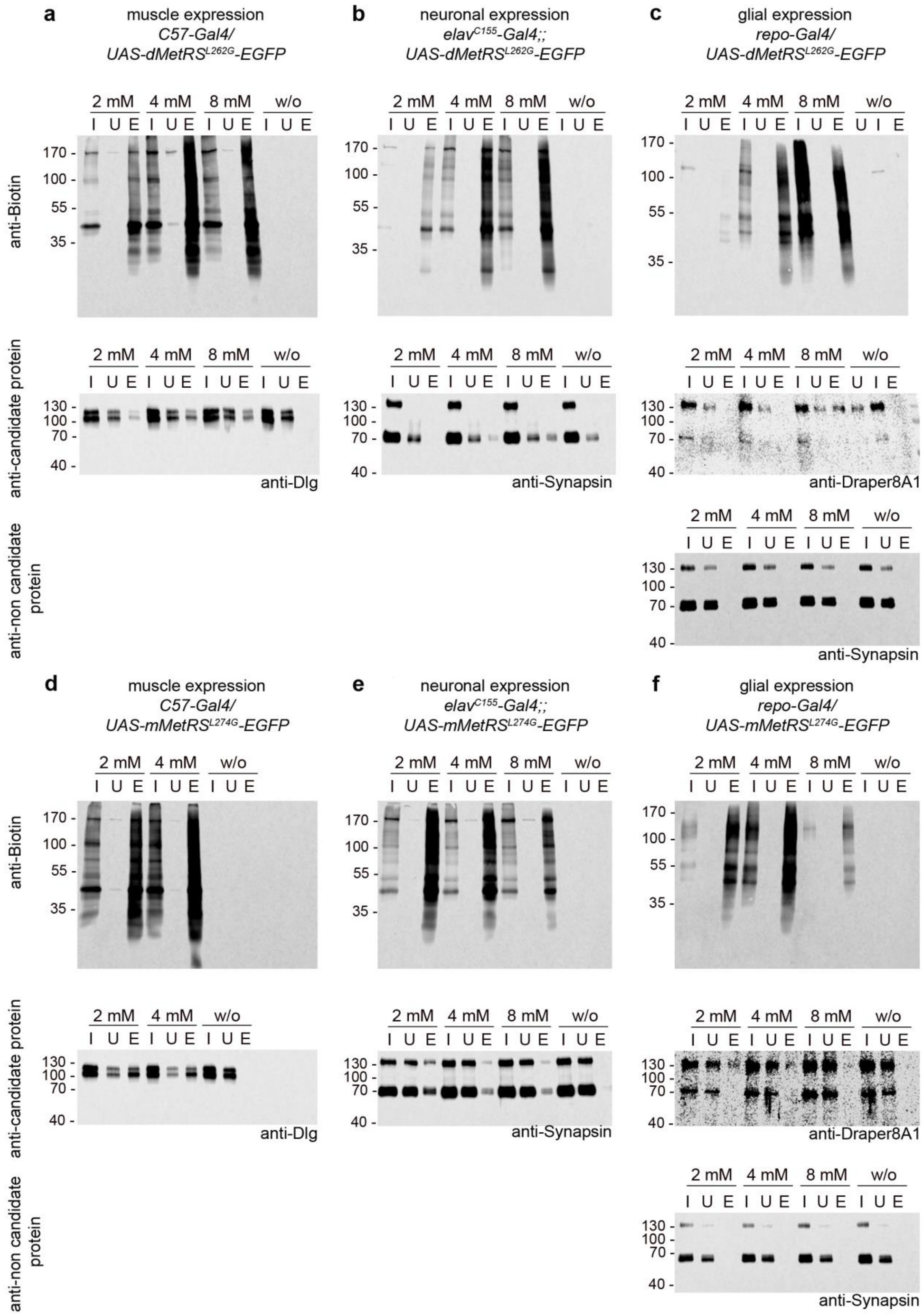

Chronic ANL incorporation into muscle, neuronal and glial proteins with varying ANL concentrations. L3 stage larval body walls (*a, d*) were dissected after chronic ANL feeding. Heads from adult flies (*b, c, e, f*) were collected 0-3 days post-eclosion after chronic ANL feeding using different concentrations of ANL as indicated. All tissue samples were lysed, clicked to a Biotin alkyne tag and purified using NeutrAvidin agarose. Depicted are representative immunoblots showing input (I, before NeutrAvidin purification), unbound (U, no ANL-containing proteins), and eluate fractions (E, enriched ANL-labeled proteins after NeutrAvidin purification) at the global protein level (anti-Biotin) and for selected candidate proteins. For dMetRS^{L262G}-EGFP (*a–c)* increasing ANL concentrations resulted in increased signal intensity for ANL-harboring proteins on the global protein level (*a-c*, *"anti-Biotin"*) as well as for selected candidate proteins such as Dlg, Synapsin or Draper I (*a-c,* "anti-candidate protein") when larvae or flies expressed dMetRS^{L262G}-EGFP either in larval muscle cells (*a*), in neurons (*b*) or glia cells (*c*) of adult flies. With larval expression of mMetRS^{L274G}-EGFP in muscle cells (*d*) high levels of ANL incorporation were reached already at 2 mM ANL as depicted for the global protein level (*d,* "anti-Biotin") and for Dlg (*d,* "anti-candidate protein"). Expression of mMetRS^{L274G}-EGFP in neurons (*e*) even showed more intense labeling at 2 mM as compared to higher concentrations of 4 mM or 8 mM ANL both on the global protein level (*e,* "anti-Biotin") and for the candidate protein Synapsin (*e, "*anti-candidate protein"). Note, that no ANL-containing Synapsin could be detected in any of the samples derived from animals expressing either MetRS^{LtoG} in glial cells (*c, f,* "anti-non candidate protein") demonstrating the specificity of this metabolic labeling approach.

Supplementary Figure 6:

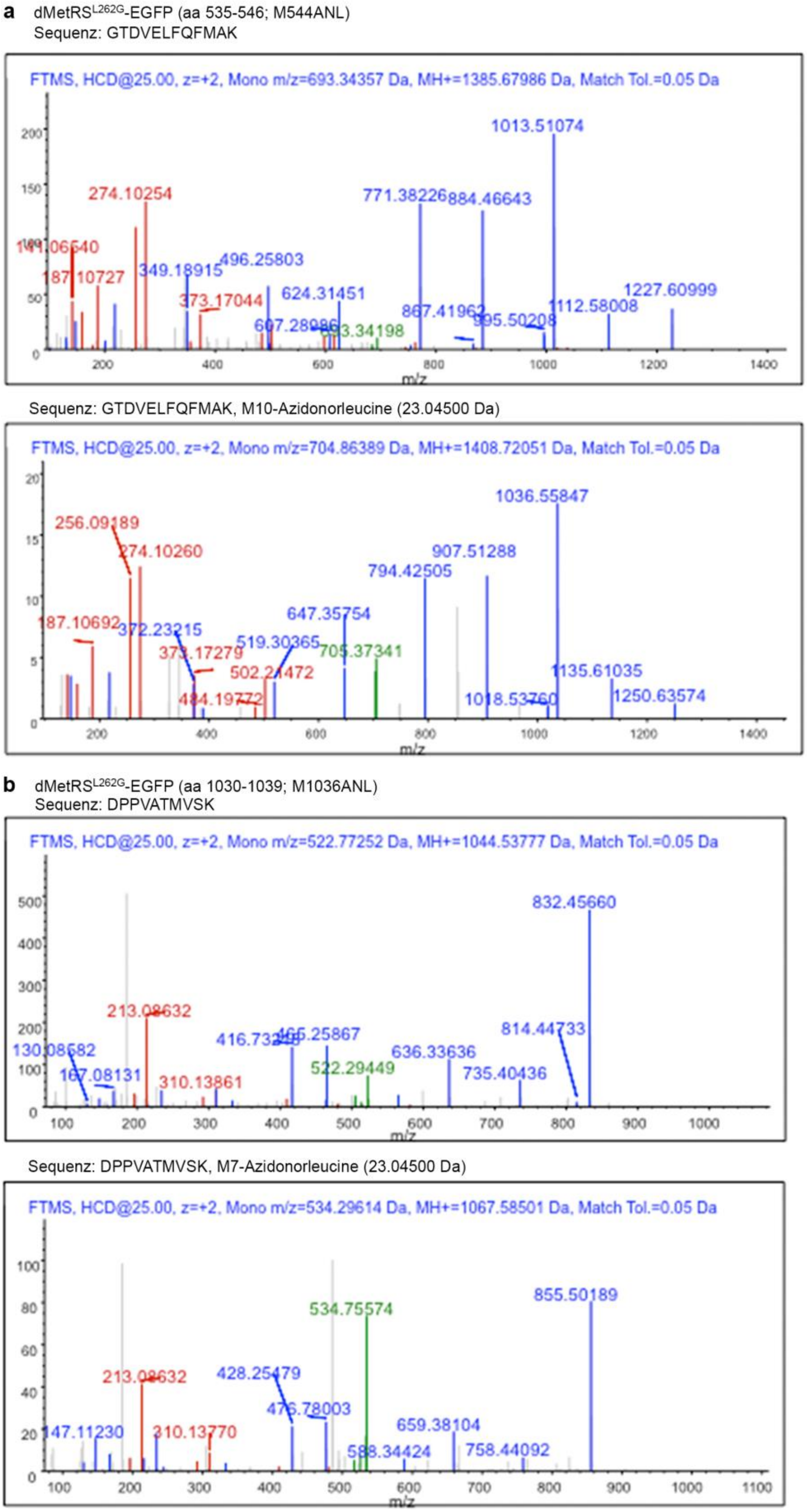

MetRS^{LtoG} incorporates ANL instead of internal methionine residues. MS/MS spectra of two internal dMetRS^{L262G}-EGFP peptides (*a*, aa 535-546; *b*, aa 1030-1039) identified from ANL-labeled dMetRS^{L262G}-EGFP purified from larval body walls after

chronic treatment with 4 mM ANL. b ions are marked in red and y ions in blue. Shown are in the upper panels the unmodified peptides and in the lower panels the two ANL-modified ones. All identified peptides were filtered with 1% FDR (false discovery rate), top rank, mass accuracy, and a minimum of 3 identified peptides. Note, that the ratio of ANL-labeled to unlabeled peptide is 1:10.

Supplementary Figure 7:

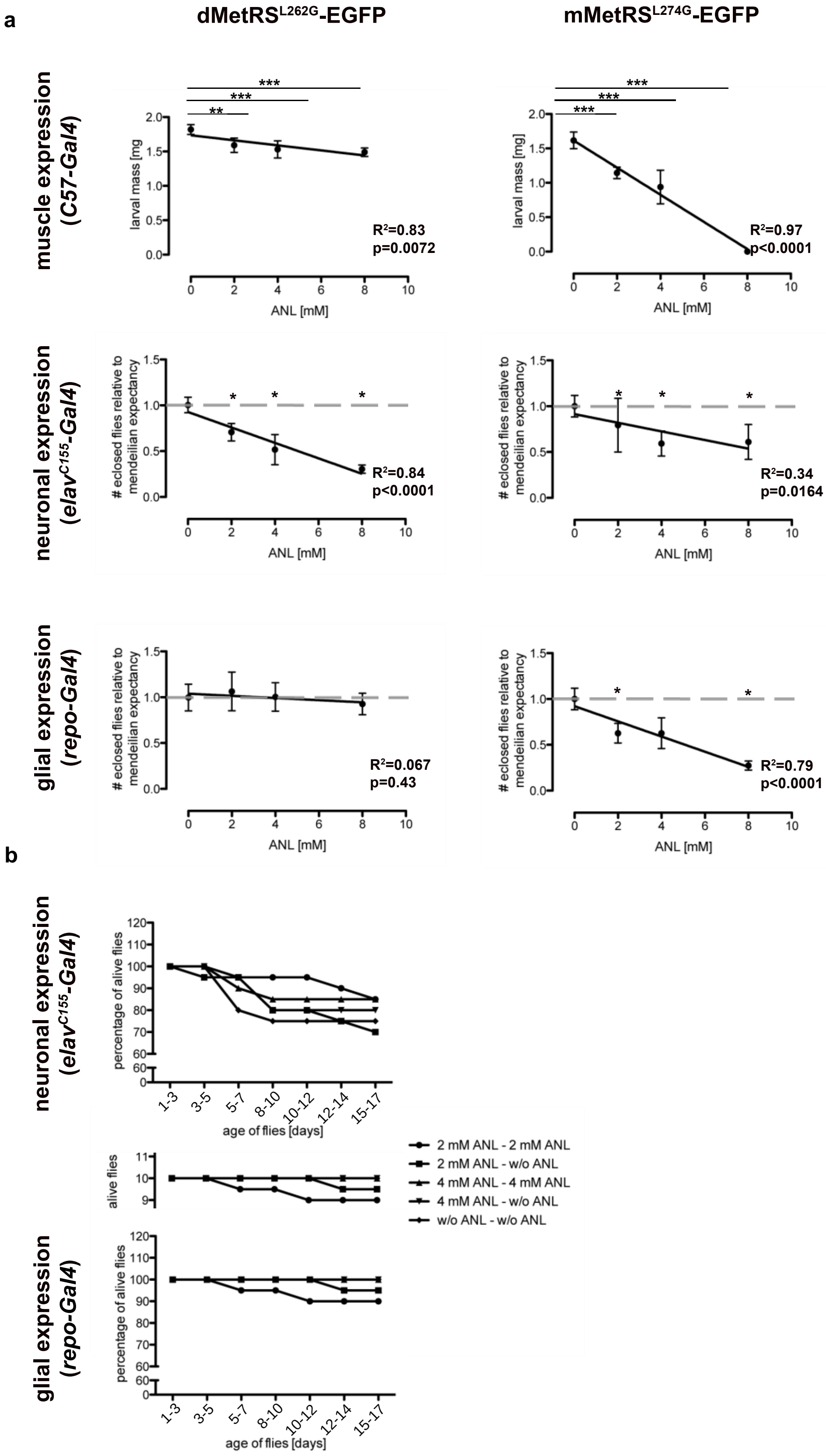

Concentration-dependent toxicity of ANL incorporation in larvae and adult flies. (*a*) Effects of ANL-incorporation into muscle proteomes were assessed by

determining the body weights of 3rd instar larvae expressing $MetRS^{LtoG}$ under control of *C57-Gal4*. ANL incorporation by $dMetRS^{L262G}$-EGFP leads to a moderate reduction of body weight. A more pronounced reduction was observed for $mMetRS^{L274G}$-EGFP with 8 mM ANL leading to larval lethality (*a*; ONE-way ANOVA with Dunnett post hoc test, ***: $p<0.001$, n=3, 4-12 larvae/condition). Body weights of $dMetRS^{L262G}$-EGFP or $mMetRS^{L274G}$-EGFP expressing larvae correlate with administered ANL concentration (*C57-Gal4/UAS-dMetRS^{L262G}-EGFP*: R^2=0.83, p=0.0072; *C57-Gal4/UAS-mMetRS^{L274G}-EGFP*: R^2=0.97, P<0.0001, n=3). Effects of ANL incorporation into neuronal and glial proteomes was assessed by determining eclosion rates of adults upon *elav^{C155}*- or *repo-Gal4*-driven $MetRS^{LtoG}$ expression (number of progeny: 51-183); theoretical mean indicated by dashed line). Eclosion of $dMetRS^{L262G}$-EGFP and $mMetRS^{L274G}$-EGFP expressing flies was significantly reduced for neuronal $dMetRS^{L262G}$-EGFP in presence of 2 mM, 4 mM and 8 mM ANL (one sample t-Test, *:$p<0.05$, *elav^{C155}-Gal4/UAS-dMetRS^{L262G}-EGFP*: 2 mM ANL: p=0.0374, 4 mM ANL: p=0.0351, 8 mM ANL: p=0.0017, *elav^{C155}-Gal4/UAS-mMetRS^{L274G}-EGFP:* 2 mM ANL: p=0.0369, 4 mM ANL: p=0.0099, 8 mM ANL: p=0.0262, n=3). Eclosion rate of neuronal $dMetRS^{L262G}$-EGFP or $mMetRS^{L274G}$-EGFP expressing flies correlates with increasing ANL concentration (*elav^{C155}-Gal4;;UAS-dMetRS^{L262G}-EGFP*: R^2=0.84, p<0.0001, *elav^{C155}-Gal4;;UAS-mMetRS^{L274G}-EGFP*: R^2=0.34, p=0.0164, n=4). For glial expression of $MetRS^{LtoG}$ effects on the eclosion rate were only monitored in case of $mMetRS^{L274G}$-EGFP at 2 mM ANL and 8 mM ANL (one sample t-Test, *:$p<0.05$, p=0.002, n=3; R^2=0.79, p<0.0001). No correlation between ANL concentrations and eclosion rates were observed for flies expressing $dMetRS^{L262G}$-EGFP in glia cells (*repo-Gal4/UAS-dMetRS^{L262G}-EGFP*: R^2=0.067, p=0.43, n=3). Error bars depict SD. (*b*) Survival rate of adult flies during neuronal or glial incorporation of ANL. Crosses between *elav^{C155}*- or *repo-Gal4*- lines and *UAS-dMetRS^{L262G}-EGFP* effectors were reared on ONM containing either 0, 2 mM, or 4 mM ANL. One to three day old adult progeny flies (5 female, 5 male) were transferred onto ONM with or without ANL (ANL concentrations during larval/pupal development - post-eclosion as indicated). A control group was reared on ANL-free ONM. The number of alive flies was checked every second day. No discernible ANL effects on the survival rates of adults became evident under these conditions (n=2).

Supplementary Figure 8:

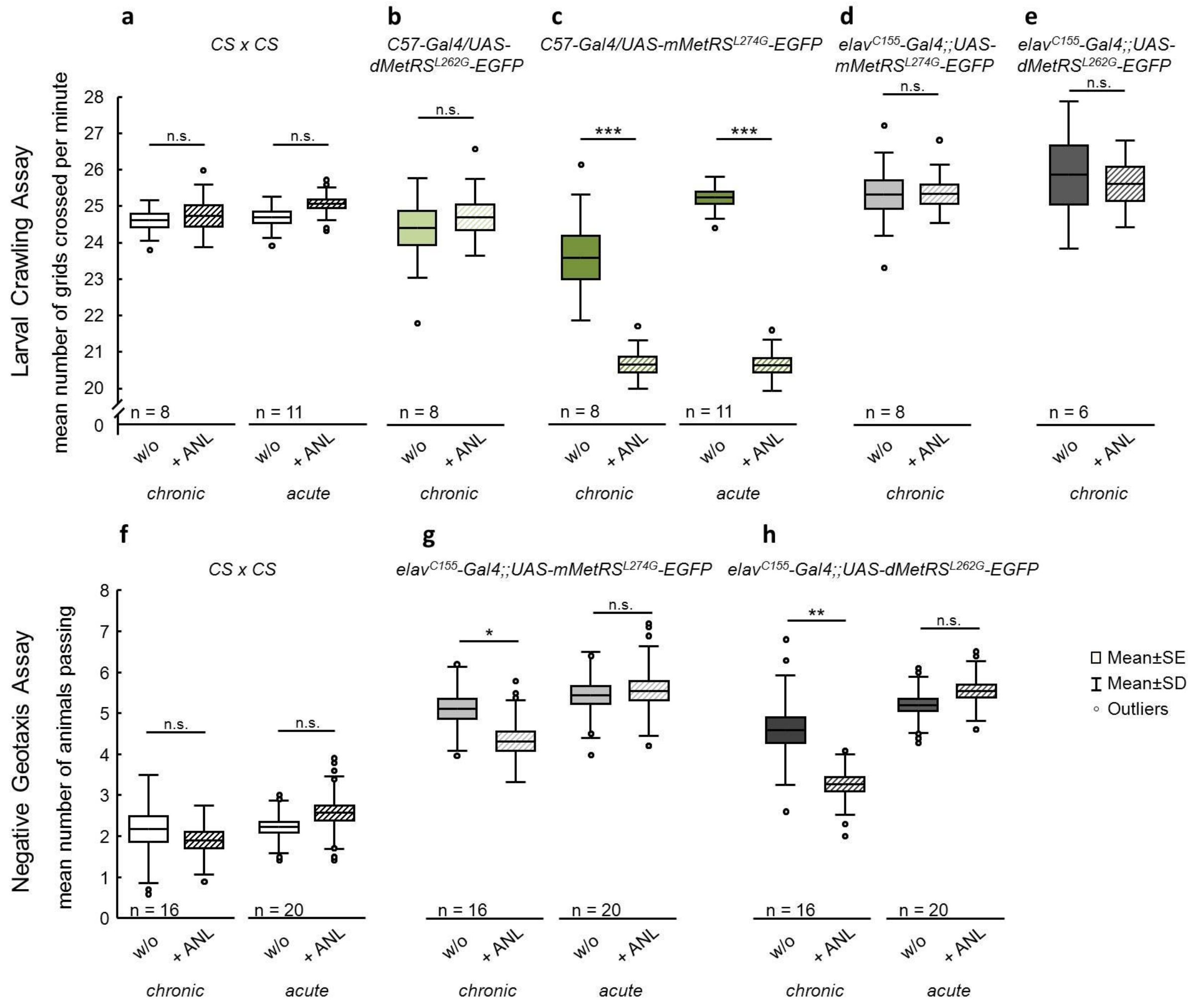

Limited behavioral effects after acute ANL feeding. (*a-e*) Larval crawling assay to determine differences in locomotion after chronic or acute (24 h) ANL feeding. Shown is the mean number of grid lines larvae crossed within one minute (*a*) Wild type larval locomotion was not affected by chronic or acute ANL feeding. (*b*) Locomotion of *C57-Gal4*/*UAS-dMetRS*^{L262G}*-EGFP* larvae was not affected by chronic ANL feeding. (*c*) Permanent and acute mMetRS^{L274G}-EGFP-mediated incorporation of ANL into muscle proteins (*C57-Gal4*) caused impairment in larval locomotion. Locomotion of *elav*^{C155}*-Gal4*;;*UAS-mMetRS*^{L274G}*-EGFP* (*d*) and *elav*^{C155}*-Gal4*;;*UAS-dMetRS*^{L262G}*-EGFP* larvae (*e*) after chronic ANL feeding did not differ from locomotion of the respective control group reared without ANL. (*f-h*) Rapid iterative negative geotaxis assay after chronic or acute (48 h) ANL feeding. Shown is the mean number of animals that passed the 8-cm mark. (*f*) Neither chronic nor acute ANL feeding affected adult climbing ability in wild type flies. Adult climbing ability of *elav*^{C155}*-Gal4*;;*UAS-mMetRS*^{L274G}*-EGFP* (*g*) and *elav*^{C155}*-Gal4*;;*UAS-dMetRS*^{L262G}*-EGFP* flies

(*h*) was impaired after chronic ANL feeding, whereas acute ANL feeding did not affect adult climbing ability. Data are presented as mean = horizontal line, standard error of the mean = box, standard deviation = whiskers, outliers = circles. Student's t-tests were used to compare groups (n=6-20 as indicated in the figure). n.s. indicates not significant ($p > 0.05$), *$p < 0.05$, **$p < 0.01$, ***$p < 0.001$; statistical analyses are shown in Supplementary Table 1).

Supplementary Figure 9:

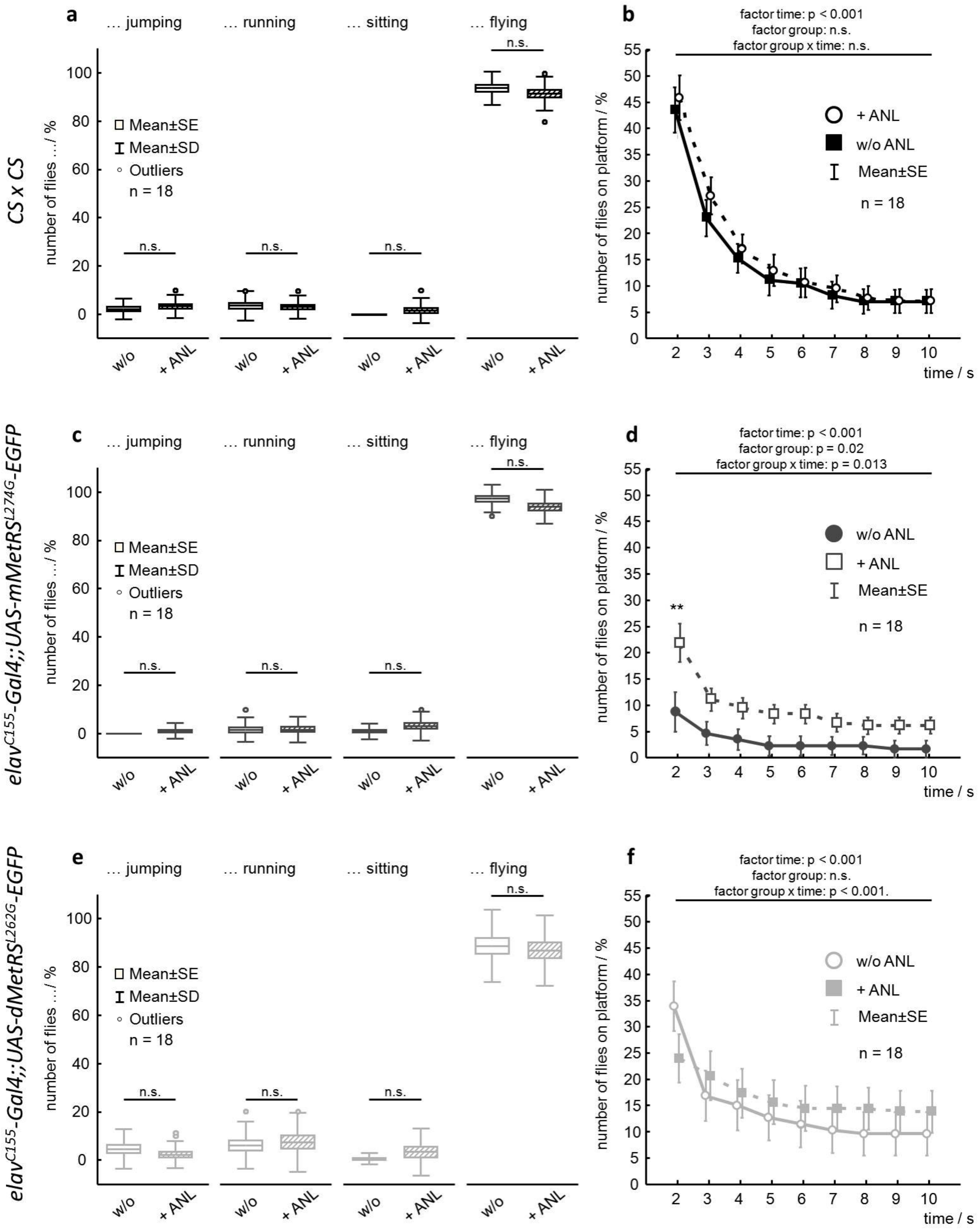

Limited effects on adult locomotion after chronic ANL feeding. We tested adult locomotion in the island assay by assessing the percentage of flies jumping, running, sitting, or flying after being released on a platform (mean = horizontal line, standard error of the mean = box, standard deviation = whiskers, outliers = circles). In addition, the mean time to clear the platform was determined (whiskers = standard error of the

mean). Chronic ANL feeding did not affect the percentage of flies jumping, running, sitting, or flying in wild type flies (*a*), *elav^{C155}-Gal4;;UAS-mMetRS^{L274G}-EGFP* flies (*c*) or *elav^{C155}-Gal4;;UAS-dMetRS^{L262G}-EGFP* flies (*e*). The time to clear the platform did not differ between flies raised on non-ANL supplemented food and flies raised on food supplemented with 4 mM ANL for wild type flies (*b*) and *elav^{C155}-Gal4;;UAS-dMetRS^{L262G}-EGFP flies* (*f*). The time to clear the platform, however, was extended after permanent ANL feeding for *elav^{C155}-Gal4;;UAS-mMetRS^{L274G}-EGFP* flies (*d*). Student's t-tests (*a, c, e*) or repeated measurement ANOVAs (*b, d, f*) were used to compare groups (n=18). n.s. indicates not significant ($p > 0.05$); statistical analyses are shown in Supplementary Table 2).

Supplementary Figure 10:

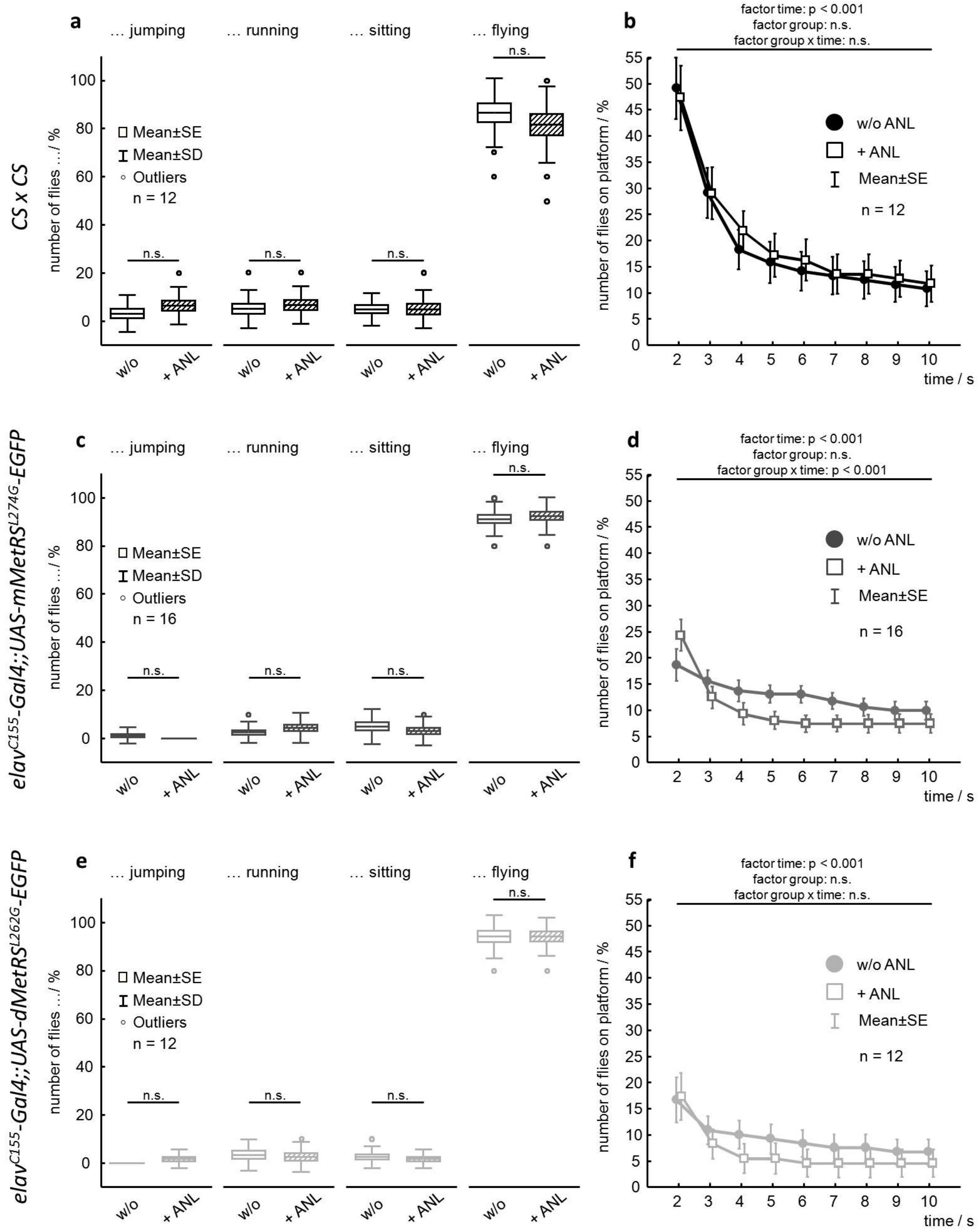

No effects on adult locomotion after acute ANL feeding. We tested adult locomotion in the island assay by assessing the percentage of flies jumping, running, sitting, or flying after being released on a platform (mean = horizontal line, standard error of the mean = box, standard deviation = whiskers, outliers = circles). In addition, the mean time to clear the platform was determined (whiskers = standard error of the

mean). Acute ANL feeding (48 h) did not affect the percentage of flies jumping, running, sitting, or flying in wild type flies (*a*), *elav*^{C155}*-Gal4;;UAS-mMetRS*^{L274G}*-EGFP* flies (*c*) or *elav*^{C155}*-Gal4;;UAS-dMetRS*^{L262G}*-EGFP* (*e*). The time to clear the platform for wild type flies (*b*), *elav*^{C155}*-Gal4;;UAS-mMetRS*^{L274G}*-EGFP* flies (*d*), and *elav*^{C155}*-Gal4;;UAS-dMetRS*^{L262G}*-EGFP flies* (*f*) did not differ between flies fed on non-ANL supplemented food and flies fed on food supplemented with 4 mM ANL. Student's t-tests (*a, c, e*) or repeated measurement ANOVAs (*b, d, f*) were used to compare groups (n=12-18 as indicated in the figure). n.s. indicates not significant ($p > 0.05$); statistical analyses are shown in Supplementary Table 3).

Supplementary Figure 11:

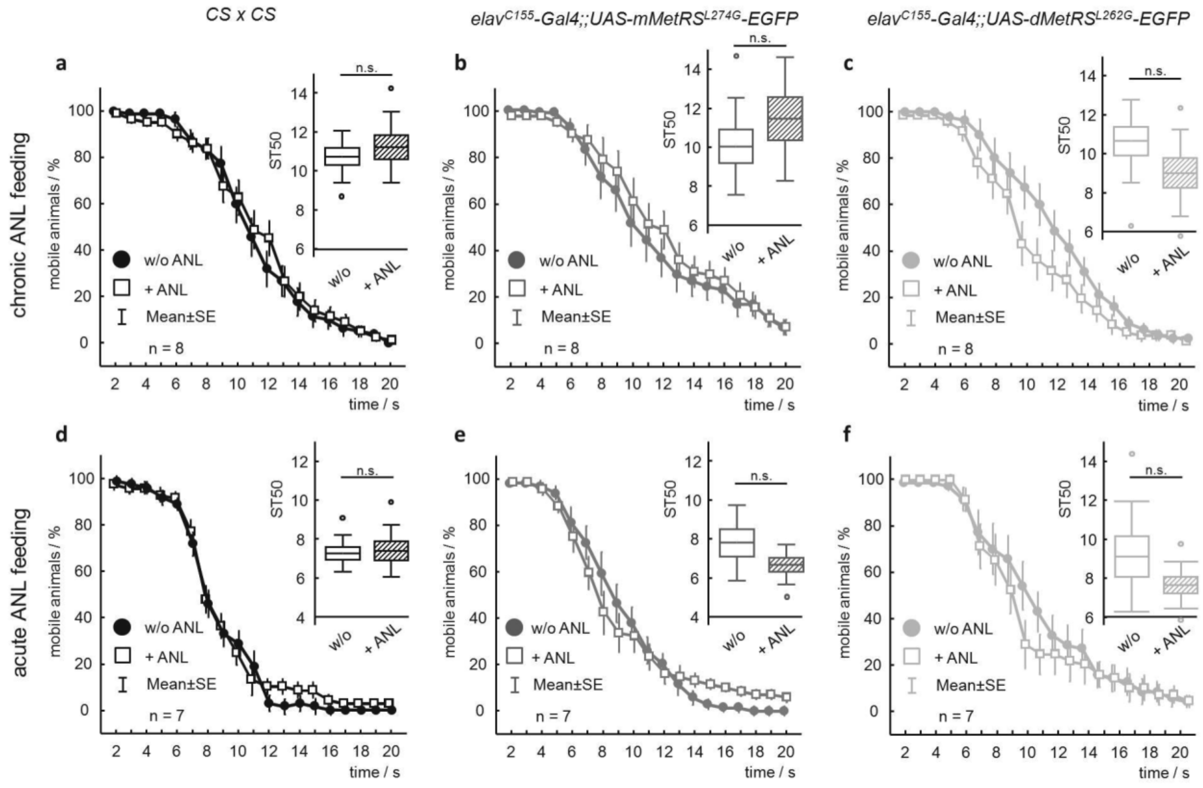

No effects on ethanol sensitivity after chronic or acute ANL feeding. We tested resistance to toxins with an ethanol sensitivity assay. Vials containing 10 flies were sealed with ethanol moistened plugs and videotaped for 20 minutes. The number of mobile flies was determined every minute. Shown is the mean number of mobile flies over the time course of 20 minutes (whiskers = standard error of the mean). The inlet shows half-maximal sedation (ST50) (mean = horizontal line, standard error of the mean = box, standard deviation = whiskers, outliers = circles). Neither chronic nor acute (48 h) ANL feeding affected the mean number of mobile wild type (*a*, *d*), *elav^{C155}-Gal4;;UAS-mMetRS^{L274G}-EGFP (b, e), or elav^{C155}-Gal4;;UAS-dMetRS^{L262G}-EGFP (c, f)* flies over the time course of 20 minutes. Similarly, the mean ST50 was not affected for either of the genotypes after chronic or acute ANL feeding. Student's t-tests were used to compare ST50. Repeated measurement ANOVAs were used to compare the mean number of mobile flies over the time course of 20 minutes (n=7-8 as indicated in the figure). n.s. indicates not significant ($p > 0.05$); statistical analyses are shown in Supplementary Table 4).

Supplementary Figure 12:

Uncropped Blot Figure 2 a
(“anti-candidate protein“, anti-Dlg)

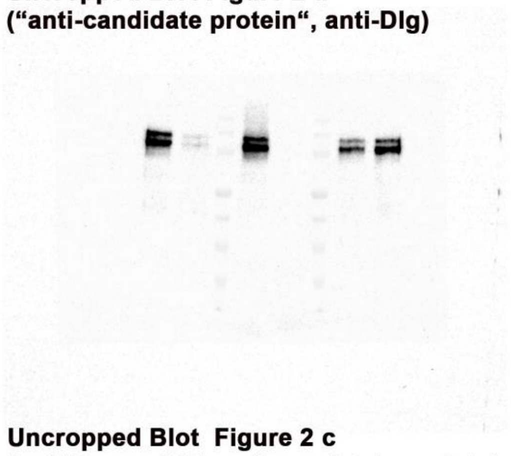

Uncropped Blot Figure 2 c
(anti-Draper 8A1, anti-candidate protein)

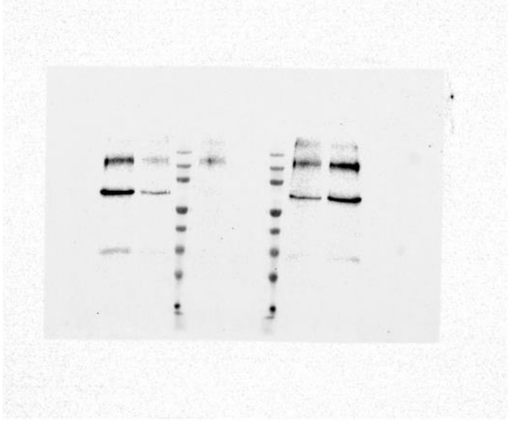

Uncropped Blot Figure 2 c
(anti-Dlg, anti-candidate protein)

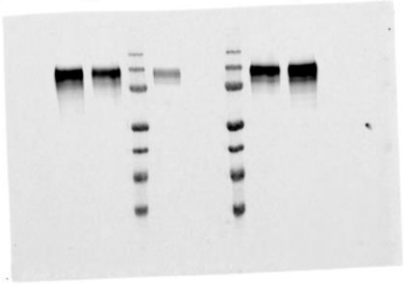

Uncropped Blot Figure 2 d

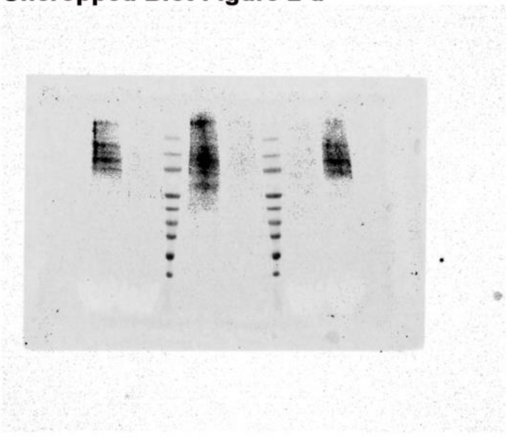

Uncropped western blots

Supplementary Table 1: Statistical analyses for the larval crawling and the RING assay

**Larval Crawling Assay**		**mean number of grids crossed per minute**		
**Phenotype**	**Treatment**	**w/o**	**+ ANL**	**Student's t-test**
Wild type Canton S	chronic	24.61	24.74	t = -0.344; p = 0.736; df = 14
	acute	24.68	25.05	t = -1.701; p = 0.105; df = 20
C57-Gal4/UAS-dMetRS^{L262G}-EGFP	chronic	24.43	24.73	t = -0.492; p = 0.631; df = 14
C57-Gal4/UAS-mMetRS^{L274G}-EGFP	chronic	23.56	20.63	t = 4.491; p < 0.001; df = 14
	acute	25.23	20.63	t = 16.682; p < 0.001; df = 20
elav^{C155}-Gal4;; UAS-mMetRS^{L274G}-EGFP	chronic	25.33	25.34	t = -0.025; p = 0.980; df = 14
elav^{C155}-Gal4;;UAS-dMetRS^{L262G}-EGFP	chronic	25.82	25.57	t = 0.261; p = 0.800; df = 10
**RING Assay**		**mean number of animals passing**		
**Phenotype**	**Treatment**	**w/o**	**+ ANL**	**Student's t-test**
Wild type Canton S	chronic	2.19	1.91	t = 0.705; p = 0.486; df = 30
	acute	2.23	2.58	t = -1.405; p = 0.168; df = 38
elav^{C155}-Gal4;;UAS-mMetRS^{L274G}-EGFP	chronic	5.13	4.33	t = 2.228; p = 0.034; df = 30
	acute	5.45	5.55	t = -0.310; p = 0.758; df = 38
elav^{C155}-Gal4;;UAS-dMetRS^{L262G}-EGFP	chronic	4.59	3.27	t = -3.469; p = 0.002; df = 30
	acute	5.21	5.55	t = -1.511; p = 0.139; df = 38

Supplementary Table 2: Statistical analyses for the island assay after chronic ANL feeding

Island Assay chronic	mean number of animals …			
Phenotype		w/o	+ ANL	Student's t-test
Wild type Canton S	...jumping	2.22	3.33	t = -0.729; p = 0.471; df = 34
	...running	3.89	3.33	t = 0.303; p = 0.766; df = 34
	...sitting	0.00	1.67	t = -1.374; p = 0.178; df = 34
	...flying	93.89	91.67	t = 0.949; p = 0.349; df = 34
	**time to clear the platform:**			rm ANOVA: time: $F_{8;264}$ = 75.808, p < 0.001; group: $F_{1;33}$ = 0.194, p = 0.663; group x time: $F_{8;264}$ = 0.236, p = 0.984.
elav^{C155}-Gal4;;UAS-mMetRS^{L274G}-EGFP	**mean number of animals …**			
		**w/o**	**+ ANL**	**Student's t-test**
	...jumping	0.00	1.11	t = -1.458; p = 0.154; df = 34
	...running	1.67	1.73	t = -0.036; p = 0.972; df = 34
	...sitting	1.11	3.33	t = -1.394; p = 0.172; df = 34
	...flying	97.22	93.83	t = 1.588; p = 0.122; df = 34
	**time to clear the platform:**			rm ANOVA: time: $F_{8;264}$ = 16.482, p < 0.001; group: $F_{1;33}$ = 6.014, p = 0.02; group x time: $F_{8;264}$ = 2.474, p = 0.013.
elav^{C155}-Gal4;;UAS-dMetRS^{L262G}-EGFP	**mean number of animals … / %**			
		**w/o**	**+ ANL**	**Student's t-test**
	...jumping	4.57	2.28	t = 0.984; p = 0.332; df = 34
	...running	6.11	7.47	t = -0.368; p = 0.715; df = 34
	...sitting	0.56	3.40	t = -1.201; p = 0.238; df = 34
	...flying	88.77	86.85	t = 0.388; p = 0.700; df = 34
	**time to clear the platform:**			rm ANOVA: time: $F_{8;264}$ = 44.04, p < 0.001; group: $F_{1;33}$ = 0.134, p = 0.716; group x time: $F_{8;264}$ = 7.438, p < 0.001.
				Tukey HSD post hoc test for group x time interaction: At no time point a significant difference between groups was determined.

Supplementary Table 3: Statistical analyses for the island assay after acute ANL feeding

Island Assay acute	mean number of animals …			
Phenotype		w/o	+ ANL	Student's t-test
Wild type Canton S	...jumping	3.33	6.67	t = -1.049; p = 0.306; df = 22
	...running	5.00	6.67	t = -0.518; p = 0.61; df = 22
	...sitting	5.00	5.00	t = 0.000; p = 1.000; df = 22
	...flying	86.67	81.67	t = 0.81; p = 0.427; df = 22
	**time to clear the platform:**			rm ANOVA: time: $F_{8;168}$ = 55.402, p < 0.001; group: $F_{1;21}$ = 0.036, p = 0.852; group x time: $F_{8;168}$ = 0.217, p = 0.988.
elav^{C155}-Gal4;;UAS-mMetRS^{L274G}-EGFP	**mean number of animals …**			
		**w/o**	**+ ANL**	**Student's t-test**
	...jumping	1.25	0.00	t = -1.464; p = 0.154; df = 30
	...running	2.50	4.38	t = 0.972; p = 0.339; df = 30
	...sitting	5.00	3.13	t = -0.792; p = 0.434; df = 30
	...flying	91.25	92.50	t = 0.473; p = 0.64; df = 30
	**time to clear the platform:**			rm ANOVA: time: $F_{8;240}$ = 28.217, p < 0.001; group: $F_{1;30}$ = 1.374, p = 0.250; group x time: $F_{8;240}$ = 4.684, p < 0.001.
				Tukey HSD post hoc test for group x time interaction: At no time point a significant difference between groups was determined.
elav^{C155}-Gal4;;UAS-dMetRS^{L262G}-EGFP	**mean number of animals …**			
		**w/o**	**+ ANL**	**Student's t-test**
	...jumping	0.00	1.67	t = -1.483; p = 0.152; df = 22
	...running	3.33	2.50	t = 0.321; p = 0.752; df = 22
	...sitting	2.50	1.67	t = 0.484; p = 0.633; df = 22
	...flying	94.17	94.17	t = 0.000; p = 1.000; df = 22
	**time to clear the platform:**			rm ANOVA: time: $F_{8;168}$ = 12.350, p < 0.001; group: $F_{1;21}$ = 0.547, p = 0.468; group x time: $F_{8;168}$ =

		0.510, p = 0.847.

Supplementary Table 4: Statistical analyses for the ethanol sensitivity assay

**Ethanol Sensitivity Assay**		**mean ST50 / min**		
**Phenotype**	**Treatment**	**w/o**	**+ ANL**	**Student's t-test**
Wild type Canton S	chronic	10.74	11.21	t = 0.596; p = 0.561; df = 14
	acute	7.28	7.40	t = 0.196; p = 0.848; df = 12
	**mobility time course chronic:**			rm ANOVA: time: $F_{18;252}$ = 216.828, p < 0.001; group: $F_{1;14}$ < 0.001, p = 0.992; group x time: $F_{18;252}$ = 0.779, p = 0.724.
	**mobility time course acute:**			rm ANOVA: time: $F_{18;216}$ = 264.620, p < 0.001; group: $F_{1;12}$ = 1.459, p = 0.250; group x time: $F_{18;216}$ = 0.568, p = 0.92.
elav^{C155}-Gal4;;UAS-mMetRS^{L274G}-EGFP		**mean ST50 / min**		
	**Treatment**	**w/o**	**+ ANL**	**Student's t-test**
	chronic	10.07	11.48	t = 0.988; p = 0.340; df = 14
	acute	7.81	6.69	t = 1.359; p = 0.199; df = 12
	**mobility time course chronic:**			rm ANOVA: time: $F_{18;252}$ = 151.628, p < 0.001; group: $F_{1;14}$ = 0.189, p = 0.670, group x time: $F_{18;252}$ = 0.727, p = 0.782.
	**mobility time course acute:**			rm ANOVA: time: $F_{18;216}$ = 179.095, p < 0.001; group: $F_{1;12}$ = 0.026, p = 0.874; group x time: $F_{18;216}$ = 2.055, p = 0.008.
				Tukey HSD post hoc test for group x time interaction: At no time point a significant difference (p < 0.05) between groups was determined.
elav^{C155}-Gal4;;UAS-dMetRS^{L262G}-EGFP		**mean ST50 / min**		
	**Treatment**	**w/o**	**+ ANL**	**Student's t-test**
	chronic	10.66	9.02	t = 1.508; p = 0.154; df = 14
	acute	9.11	7.65	t = -1.254; p = 0.234; df = 12
	**mobility time course chronic:**			rm ANOVA: time: $F_{19;266}$ = 171.335, p < 0.001; group: $F_{1;14}$ = 1.885, p = 0.191; group x time: $F_{19;266}$ = 1.678, p = 0.040.
				Tukey HSD post hoc test for group x time interaction: At no time point a significant difference (p < 0.05) between groups was determined.
	**mobility time course acute:**			rm ANOVA: time: $F_{19;228}$ = 109.161, p < 0.001; group: $F_{1;12}$ = 0.394, p = 0.542; group x time: $F_{19;228}$ = 1.156, p = 0.298.

Supplementary Note 1:

We tested the efficiency of chronic ANL incorporation by dMetRS^{L262G}-EGFP and mMetRS^{L274G}-EGFP into different cellular proteomes under varying ANL concentrations (Fig. S5). We noticed that mMetRS^{L274G}-EGFP expressing cells incorporate ANL with a higher rate into the respective proteomes compared to dMetRS^{L262G}-EGFP. This increased incorporation rate however, can affect the vitality of the animals and ultimately, affect translation rates of sensitive cell types such as neurons especially under high ANL feeding conditions. We, therefore, recommend the use of either dMetRS^{L262G}-EGFP or mMetRS^{L274G}-EGFP depending on the question and nature of the experiment.

Supplementary Reference

1 Serre, L. *et al.* How methionyl-tRNA synthetase creates its amino acid recognition pocket upon L-methionine binding. *J Mol Biol* **306**, 863-876, doi:10.1006/jmbi.2001.4408 (2001).
